# Effect of carbohydrate-restricted diets and intermittent fasting on obesity, type 2 diabetes mellitus, and hypertension management: consensus statement of the Korean Society for the Study of obesity, Korean Diabetes Association, and Korean Society of Hypertension

**DOI:** 10.1186/s40885-022-00207-4

**Published:** 2022-06-01

**Authors:** Jong Han Choi, Yoon Jeong Cho, Hyun-Jin Kim, Seung-Hyun Ko, Suk Chon, Jee-Hyun Kang, Kyoung-Kon Kim, Eun Mi Kim, Hyun Jung Kim, Kee-Ho Song, Ga Eun Nam, Kwang Il Kim, Committee of Clinical Practice Guidelines, Korean Society for the Study of Obesity (KSSO), Committee of Clinical Practice Guidelines and Committee of Food and Nutrition, Korean Diabetes Association (KDA), Policy Committee of Korean Society of Hypertension (KSH), Policy Development Committee of National Academy of Medicine of Korea (NAMOK)

**Affiliations:** 1 Division of Endocrinology and Metabolism, Department of Internal Medicine, Konkuk University Medical Center, Konkuk University School of Medicine, Seoul, 05030 South Korea; 2 Department of Family Medicine, Daegu Catholic University School of Medicine, Daegu, South Korea; 3 Division of Cardiology, Department of Internal Medicine, Hanyang University College of Medicine, Seoul, South Korea; 4 Division of Endocrinology and Metabolism, Department of Internal Medicine, St. Vincent’s Hospital, College of Medicine, The Catholic University of Korea, Seoul, South Korea; 5 Department of Endocrinology and Metabolism, Kyung Hee University College of Medicine, Seoul, South Korea; 6 Department of Family Medicine, Konyang University College of Medicine, Daejeon, South Korea; 7 Department of Family Medicine, Gachon University College of Medicine, Incheon, South Korea; 8 Department of Dietetics, Kangbuk Samsung Hospital, Seoul, South Korea; 9 Institute for Evidence-based Medicine, Cochrane Korea, Department of Preventive Medicine, Korea University College of Medicine, Seoul, South Korea; 10 Department of Family Medicine, Korea University Guro Hospital, Korea University College of Medicine, Seoul, 08308 Republic of Korea; 11 Division of Geriatrics, Department of Internal Medicine, Seoul National University Bundang Hospital, Seongnam, 13620 Republic of Korea

**Keywords:** Obesity, Diabetes mellitus, Hypertension, Diet, Carbohydrate

## Abstract

**Background:**

Carbohydrate-restricted diets and intermittent fasting (IF) have been rapidly gaining interest among the general population and patients with cardiometabolic disease, such as overweight or obesity, diabetes, and hypertension. However, there are limited expert recommendations for these dietary regimens. This study aimed to evaluate the level of scientific evidence on the benefits and harms of carbohydrate-restricted diets and IF to make responsible recommendations.

**Methods:**

A meta-analysis and systematic literature review of 66 articles on 50 randomized controlled clinical trials (RCTs) of carbohydrate-restricted diets and ten articles on eight RCTs of IF was performed.

**Results:**

Based on the analysis, the following recommendations are suggested. In adults with overweight or obesity, a moderately-low carbohydrate or low carbohydrate diet (mLCD) can be considered as a dietary regimen for weight reduction. In adults with type 2 diabetes, mLCD can be considered as a dietary regimen for improving glycemic control and reducing body weight. In contrast, a very-low carbohydrate diet (VLCD) and IF are recommended against in patients with diabetes. Furthermore, no recommendations are suggested for VLCD and IF in adults with overweight or obesity, and carbohydrate-restricted diets and IF in patients with hypertension.

**Conclusion:**

Here, we describe the results of our analysis and the evidence for these recommendations.

**Supplementary Information:**

The online version contains supplementary material available at 10.1186/s40885-022-00207-4.

## Introduction

Obesity, type 2 diabetes mellitus (T2DM), and hypertension are the most important risk factors for cardiovascular disease and are the most common causes of morbidity and mortality [1–4]. Structured dietary intervention plays a crucial role in preventing and managing these cardiometabolic diseases. Major clinical practice guidelines commonly recommend losing more than 5% of body weight and reducing total caloric intake [5–8]. Nevertheless, reducing total caloric intake enough to lose weight requires tremendous effort, and maintaining it over the long term is much more challenging. Alternatively, carbohydrate-restricted diets and intermittent fasting (IF) are emerging as relatively easy and effective popular dietary regimens for reducing body weight [9].

Carbohydrates make up more than half of an individual’s calorie intake, and restricting them can be critical in reducing total calories and body weight [10]. Obesity, T2DM, and hypertension constitute metabolic syndrome, and glucose-stimulated hyperinsulinemia and insulin resistance are major contributors to the pathogenesis of these diseases [11]. Reducing carbohydrate that is absorbed in the form of glucose or fructose and leading to immediate hyperglycemia may help prevent and improve these conditions [12]. However, clinical evidence on the benefits and harms of carbohydrate-restricted diets in these diseases remains insufficient [5–8].

IF is a generic term for a variety of eating methods involving fasting for different periods, such as several hours a day, 1 day every several days, or several days a week [13]. In some studies, IF is known to be effective in preventing diabetes, cardiovascular disease, cancer, and degenerative brain disease, as well as weight loss in overweight or obese people [14]. However, these studies were conducted for a short period with a small number of subjects, and results were heterogeneous among studies [14]. Moreover, since most studies evaluated effects in healthy adults, applying the impact on patients with cardiometabolic diseases such as morbid obesity, diabetes, and hypertension is challenging [15].

For these reasons, the principal clinical practice guidelines for managing obesity, T2DM, and hypertension do not provide specific recommendations for carbohydrate-restricted diets or IF [5–8]. Notably, the dietary approaches that lack evidence for benefits but are potentially harmful are rapidly spreading to the public without clear guidance from experts. Therefore, we aimed to conduct a meta-analysis and systematic literature review to examine the benefits and harms of these dietary regimens in adults with obesity, T2DM, and hypertension and develop recommendations based on the high-level evidence by the results.

## Methods

A systematic literature search was performed by a professional librarian for the meta-analysis with the key question “Are carbohydrate-restricted diets or IF helpful in the management of patients with overweight or obesity, diabetes, and hypertension?”. MEDLINE (via PubMed), EMbase, Cochrane, and KoreaMed databases were used, and among the literature published in English and Korean from January 1, 2000, to June 8, 2021, only randomized controlled clinical trials (RCTs) that tested the effectiveness of carbohydrate-restricted diets or IF with a study period of more than 8 weeks were included. The search strategy in MEDLINE is shown in Additional file 1: Supplementary Table 1. Further, the framework of the population, intervention, comparator, and outcomes (PICO) in developing the focused question (Additional file 1: Supplementary Table 2), and literature selection and exclusion process (Additional file 1: Supplementary Fig. 1), as well as the summary of studies included in the meta-analysis consisting of 50 RCTs (66 articles) on carbohydrate-restricted diets (Additional file 1: Supplementary Table 3) [16–81], and eight RCTs (ten articles) on IF (Additional file 1: Supplementary Table 4) [82–91]), are presented in the Additional file 1: supplementary data.

In most clinical studies, carbohydrate-restricted diets are classified as moderately-low carbohydrate diets (MCD) with carbohydrates accounting for 26–45% of total caloric intake, low carbohydrate diets (LCD) with carbohydrates accounting for 10–25%, and very-low carbohydrate diets (VLCD) with carbohydrates accounting for less than 10% [92]. Considering that the average carbohydrate intake rate is about 65% in South Korea, which is significantly higher than that of other countries, and that the greater the restriction, the lower the adherence [93]. Thus, we evaluated MCD and LCD as a combined category, moderately-low carbohydrate or low carbohydrate diets (mLCD) (Additional file 1: Supplementary Table 5). IF includes several different dietary regimens, such as time-restricted feeding, alternate-day fasting, and intermittent energy restriction, as well as those with similar meanings. The comparative diets were diets with calorie restriction equivalent to those of the intervention diets, and most were included in a calorie-restricted diet and a low fat diet. Even if it was indicated as a standard diet, it was included in the analysis if the same degree of caloric restriction was achieved.

Outcome variables included anthropometric measurements like body weight, glycemic control indicators like HbA1c, and cardiovascular risk factors like blood pressure and lipid profiles. Detailed primary and secondary outcome variables will be described in each recommendation for each study population. The analysis of outcome variables was performed by classifying as follows according to the duration of the intervention: for 6 months or less (9 to 24 weeks), for more than 6 months to 1 year or less (36 weeks to 52 weeks), and for more than 1 year. The quality of evidence for key outcome variables was evaluated and presented within 6 months since most of the results were performed within this period, and the recommendations were provided in a short-term period of 6 months or less.

We assessed the risk of bias in the included trials using the revised Cochrane risk-of-bias tool for RCTs [94]. Each trial was assessed by two independent observers, and any differences were resolved by a third observer. The final grade of recommendation and level of evidence for each statement was determined using the Grades of Recommendation, Assessment, Development, and Evaluation system [95]. All subcommittee members participated in the joint production of each part of the guideline, from inception to publication. When the agreement was incomplete regarding the final grade of recommendation and level of evidence, consensus from the chair, vice-chair, and two assigned reviewers of the subcommittee have made the outcome. Recommendations formulated by the subcommittees were reviewed by the entire members of all five committees from the four academic societies that participated in this study.

## Recommendation and evaluation of evidence for carbohydrate-restricted diets in adults with overweight or obesity

### Recommendation

In adults with overweight or obesity, a moderately-low carbohydrate diet or low carbohydrate diet (mLCD) can be considered a dietary regimen for weight reduction since similar or greater effects on weight loss are observed than the generally recommended diets. [Conditional recommendation, moderate quality of evidence].

**Table:** 

1. A low carbohydrate diet does not imply an extreme reduction in carbohydrate and increase in fat intake, and must not be practiced indiscriminately. 2. A low carbohydrate diet should reduce total caloric intake while avoiding an increase in the intake of saturated and trans fatty acids. 3. After considering sustainability and balance between benefits and risks, we decided not to provide a recommendation for very-low carbohydrate diets (VLCD).	

### Level of evidence

In the meta-analysis, 63 articles from 47 RCTs were included. While overweight or obesity was an inclusion criterion for the meta-analysis, obese patients with a body mass index (BMI) of ≥30 kg/m^2^ were included in many cases, and a small number of studies included patients with type 2 diabetes. Although studies were conducted in various countries, no studies were conducted in South Korea, and only few targeted Asians. However, studies conducted in China, Japan, and Taiwan, which have similar demographic characteristics to South Korea, had similar overall results. Due to the nature of the studies, in most cases, participants were not blinded, or related information was not described. Low fat (44.7%) and calorie-restricted (29.8%) diets were the most common control diets. The dropout rate of participants was within 20–30%. Although the risk of bias differed depending on the study, it was generally low (Additional file 1: Supplementary Fig. 2). If the sufficient effect (clinical decision threshold) was not reached, it was judged that there was imprecision. Since the dropout rate was high and the two groups were heterogenous, it was judged that there was indirectness, and thus, the level of evidence was downgraded and evaluated as either low or moderate (Additional file 1: Supplementary Table 6) (Table 1).

**Table 1:** Summary of finding for effects of carbohydrate-restricted diets and intermittent fasting in adults with overweight/obesity

Outcome	Illustrative comparative effect^a^ (95% CI)		No. of participants	Quality of the evidence (GRADE)
	Assumed effect (control)	Corresponding effect		
mLCD^b^				
Body weight, kg (follow-up: 8–24 weeks)	–3.74	–1.03 (–1.68 to –0.39)	3,660 (24 studies)	⊕⊕⊝⊝ Low
Body mass index, kg/m^2^ (follow-up: 8–24 weeks)	–1.5	–0.23 (–0.46 to 0.00)	2,750 (15 studies)	⊕⊝⊝⊝ Very low
Waist circumference, cm (follow-up: 12–24 weeks)	–4.83	–0.65 (–1.16 to –0.14)	2,340 (15 studies)	⊕⊕⊕⊝ Moderate
Fat mass, kg (follow-up: 12–24 weeks)	–2.92	–0.44 (–0.83 to –0.04)	2,080 (14 studies)	⊕⊕⊕⊝ Moderate
Fat-free mass, kg (follow-up: 12–24 weeks)	0.17	–0.17 (–0.49 to 0.14)	1,139 (10 studies)	⊕ ⊕ ⊝⊝ Low
Fat mass, % (follow-up: 12–24 weeks)	–2.7	0.09 (–0.45 to 0.64)	445 (4 studies)	⊕⊕⊝⊝ Low
Systolic blood pressure, mm Hg (follow-up: 8–24 weeks)	–4.0	–0.56 (–1.69 to 0.56)	2,612 (19 studies)	⊕⊕⊝⊝ Low
Diastolic blood pressure mmHg (Follow-up: 8 ~ 24 weeks)	− 2.5	− 0.69 (− 1.39 to 0.01)	2615 (19 studies)	⊕ ⊕ ⊝⊝ Low
Triglyceride mg/dL (Follow-up: 8 ~ 24 weeks)	− 11.8	− 13.76 (− 19.78 to − 7.74)	2896 (24 studies)	⊕ ⊕ ⊝⊝ Low
LDL-C, mg/dL (follow-up: 12–24 weeks)	− 4.6	2.29 (− 0.41 to 4.99)	2721 (21 studies)	⊕⊝⊝⊝ Very low
HDL-C, mg/dL (follow-up: 8–24 weeks)	− 0.8	2.61 (1.34 to 3.89)	2448 (20 studies)	⊕ ⊕ ⊕⊝ Moderate
HbA1c, % (follow-up: 8–24 weeks)	−0.2	− 0.20 (− 0.39 to − 0.01)	739 (8 studies)	⊕ ⊕ ⊝⊝ Low
Fasting insulin, μU/mL (follow-up: 12–24 weeks)	−0.9	− 0.94 (− 1.73 to − 0.16)	1855 (13 studies)	⊕⊕⊕⊝ Moderate
Fasting glucose, mg/dL (follow-up: 8–24 weeks)	− 3.1	–0.32 (–1.23 to 0.58)	2143 (17 studies)	⊕⊕⊝⊝ Low
C-reactive protein, mg/L (follow-up: 8–24 weeks)	− 0.1	− 0.34 (− 0.67 to − 0.01)	1391 (11 studies)	⊕ ⊕ ⊝⊝ Low
Adiponectin, μg/mL (follow-up: 8–24 weeks)	0.2	0.45 (0.15 to 0.76)	1356 (8 studies)	⊕ ⊕ ⊕⊝ Moderate
VLCDc^c^				
Body weight, kg (follow-up: 8–24 weeks)	−3.75	−3.67 (−4.84 to − 2.51)	1266 (14 studies)	⊕ ⊕ ⊕⊝ Moderate
Body mass index, kg/m^2^ (follow-up: 8–24 weeks)	−1.0	−1.88 (− 3.11 to −0.65)	388 (5 studies)	⊕ ⊕ ⊕⊝ Moderate
Waist circumference, cm (follow-up: 8–24 weeks)	−4.7	−4.11 (−8.70 to 0.49)	233 (2 studies)	⊕ ⊕ ⊝⊝ Low
Fat mass, kg (follow-up: 8–24 weeks)	−4.8	−3.01 (−6.29 to 0.27)	168 (3 studies)	⊕ ⊕ ⊝⊝ Low
Fat-free mass, kg (follow-up: 8–24 weeks)	−0.3	−1.05 (− 1.75 to − 0.35)	168 (3 studies)	⊕ ⊕ ⊝⊝ Moderate
Fat mass, % (follow-up: 8–24 weeks)	− 1.45	− 1.88 (−2.87 to − 0.89)	515 (4 studies)	⊕ ⊕ ⊕⊝ Moderate
Systolic blood pressure, mm Hg (follow-up: 8–24 weeks)	− 3.0	− 1.97 (− 3.68 to − 0.25)	506 (9 studies)	⊕ ⊕ ⊕⊝ Moderate
Diastolic blood pressure, mm Hg (follow-up: 8–24 weeks)	− 2.1	− 0.68 (− 1.79 to 0.44)	906 (9 studies)	⊕ ⊕ ⊝⊝ Low
Triglyceride, mg/dL (follow-up: 8–24 weeks)	− 11.9	− 21.33 (− 30.46 to − 12.21)	1059 (13 studies)	⊕ ⊕ ⊝⊝ Low
LDL-C, mg/dL (follow-up: 8–24 weeks)	–5.1	7.52 (3.34 to 11.70)	1023 (12 studies)	⊕ ⊕ ⊕⊝ Moderate
HDL-C, mg/dL (follow-up: 8–24 weeks)	0.0	4.30 (1.79 to 6.82)	1058 (13 studies)	⊕ ⊕ ⊝⊝ Low
HbA1c, % (follow-up: 8–24 weeks)	− 0.15	− 0.27 (− 0.50 to − 0.03)	354 (6 studies)	⊕ ⊕ ⊝⊝ Low
Fasting insulin, μU/mL (follow-up: 8–24 weeks)	−1.55	− 1.37 (− 2.89 to 0.15)	603 (6 studies)	⊕ ⊕ ⊝⊝ Low
Fasting glucose, mg/dL (follow-up: 8–24 weeks)	− 2.9	− 0.44 (− 2.66 to 1.78)	730 (9 studies)	⊕ ⊕ ⊝⊝ Low
C-reactive protein, mg/L (follow-up: 8–24 weeks)	− 0.2	− 0.63 (− 1.41 to 0.15)	371 (5 studies)	⊕ ⊕ ⊝⊝ Low
Adiponectin, μg/mL (follow-up: 8–24 weeks)	0.4	0.75 (0.29 to 1.21)	181 (2 studies)	⊕ ⊕ ⊝⊝ Low
Intermittent fasting^d^				
Body weight, kg (follow-up: 12–24 weeks)	− 3.62	−1.22 (−3.49 to 1.05)	554 (8 studies)	⊕⊝⊝⊝ Very low
Body mass index, kg/m^2^ (follow-up: 12–24 weeks)	−1.46	−0.49 (− 1.13 to 0.14)	380 (5 studies)	⊕ ⊕ ⊝⊝ Low
Waist circumference, cm (follow-up: 12–24 weeks)	−2.28	−1.95 (−4.09 to 0.2)	180 (3 studies)	⊕⊝⊝⊝ Very low
Fat mass, kg (follow-up: 12–24 weeks)	−1.1	−0.36 (− 0.87 to 0.16)	540 (8 studies)	⊕⊝⊝⊝ Very low
Fat-free mass, kg (follow-up: 12–24 weeks)	−3.7	− 0.67 (− 1.95 to 0.62)	540 (8 studies)	⊕⊝⊝⊝ Very low
Fat mass, % (follow-up: 12–24 weeks)	−0.9	0.27 (−0.48 to 1.01)	142 (3 studies)	⊕⊝⊝⊝ Very low
Systolic blood pressure, mm Hg (follow-up: 12–24 weeks)	−5.7	0.87 (−2.56 to 4.39)	404 (6 studies)	⊕⊝⊝⊝ Very low
Diastolic blood pressure, mm Hg (follow-up: 12–24 weeks)	−3.4	−0.16 (−2.89 to 2.56)	404 (6 studies)	⊕⊝⊝⊝ Very low
Triglyceride, mg/dL (follow-up: 12–24 weeks)	−22.0	−1.51 (− 17.06 to 14.04)	432 (6 studies)	⊕⊝⊝⊝ Very low
LDL-C, mg/dL (follow-up: 12–24 weeks)	−12.48	−0.24 (−5.08 to 4.59)	387 (5 studies)	⊕⊝⊝⊝ Very low
HDL-C, mg/dL (follow-up: 12–24 weeks)	0.0	−0.17 (−3.27 to 2.89)	432 (6 studies)	⊕⊝⊝⊝ Very low
HbA1c, % (follow-up: 12–24 weeks)	−0.31	0.11 (−0.04 to 0.26)	173 (3 studies)	⊕⊝⊝⊝ Very low
Fasting glucose, mg/dL (follow-up: 12–24 weeks)	−3.00	−0.89 (−4.30 to 2.53)	359 (5 studies)	⊕ ⊕ ⊝⊝ Low
Fasting insulin, μU/mL (follow-up: 12–24 weeks)	−2.6	−0.43 (−1.99 to 1.14)	314 (4 studies)	⊕ ⊕ ⊝⊝ Low
HOMA-IR (follow-up: 12–24 weeks)	−0.94	−0.22 (−1.48 to 1.05)	119 (2 studies)	⊕⊝⊝⊝ Very low

^a^The basis for the assumed effect is the mean change of outcomes compared to baseline in the control group across studies, and the corresponding effect (and its 95% CI) is based on the assumed effect in the comparison group

^b^mLCD for overweight/obesity: Patient or population (patients with overweight/obese), Intervention (mLCD)

^c^VLCD for overweight/obese: Patient or population (patients with overweight/obesity), Intervention (VLCD)

^d^Intermittent fasting for overweight/obesity: Patient or population (patients with overweight/obesity), Intervention (intermittent fasting)

*CI* confidence interval, *GRADE* Grading of Recommendations Assessment, Development and Evaluation, *mLCD* moderately-low carbohydrate or low carbohydrate diet, *LDL-C* low-density lipoprotein cholesterol, *HDL-C* high-density lipoprotein cholesterol, HbA1c glycosylated hemoglobin, *VLCD* very-low carbohydrate diet, *HOMA-IR* homeostatic model assessment for insulin resistance

GRADE Working Group grades of evidence: High quality (Further research is very unlikely to change our confidence in the estimate of effect); Moderate quality (Further research is likely to have an important impact on our confidence in the estimate of effect and may change the estimate); Low quality (Further research is very likely to have an important impact on our confidence in the estimate of effect and is likely to change the estimate); Very low quality (We are very uncertain about the estimate).

### Benefits (advantages)

In the meta-analysis, the primary outcomes for assessing the benefit of a carbohydrate-restricted diet in adults with overweight or obesity included body weight, BMI, waist circumference (WC), fat mass, body fat percentage, and fat-free mass. Secondary outcomes included blood pressure, lipid profile, fasting blood glucose, glycated hemoglobin (HbA1c), fasting serum insulin, adiponectin, C-reactive protein (CRP), and adverse effects. Table 1 summarizes the results of the meta-analysis and the level of evidence for each outcome of mLCD and VLCD.

#### Body weight and BMI

**Fig. 1.**
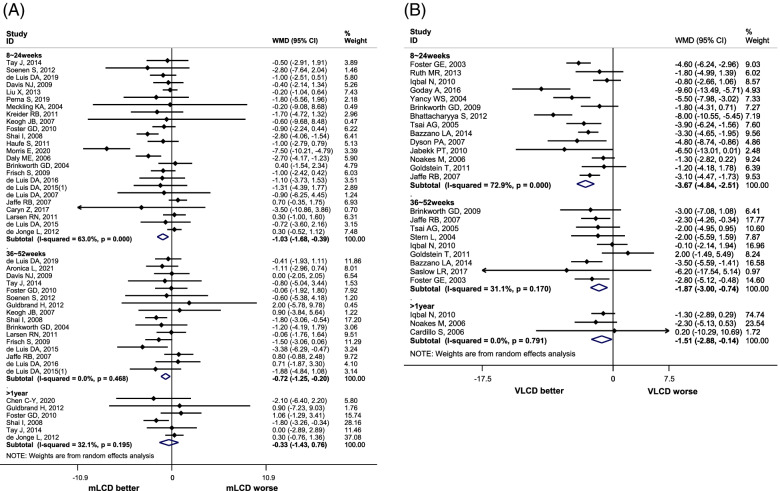
Effects of Carbohydrate-Restricted Diets on Body Weight in Adults with Overweight/Obesity. **A** Moderately-low carbohydrate or low carbohydrate diet (mLCD). **B** Very-low carbohydrate diet (VLCD)

The result of a meta-analysis of 24 articles with a study period of 6 months or less (8 weeks to 24 weeks) showed mLCD had a significant decrease in body weight (mean difference: -1.03, 95% CI: − 1.68, − 0.39), compared to the control diet (Fig. 1A). A meta-analysis of 17 studies with a study period of more than 6 months to 1 year or less (36 weeks to 52 weeks) also showed a significant decrease in body weight (mean difference: -0.72; 95% CI: − 1.25, − 0.20) compared to the control diet, but no significant weight loss was observed for more than 1 year (Fig. 1A). Compared to the control diet, VLCD significantly decreased body weight by 3.67 kg (95% CI: − 4.84, − 2.51) for the study duration of 6 months or less (8 weeks to 24 weeks), 1.87 kg (95% CI: − 3.00, − 0.74) for more than 6 months to 1 year or less (36 weeks to 52 weeks), and 1.51 kg (95% CI: − 2.88, − 0.14) for more than 1 year, but the difference in weight loss decreased as the study period increased (Fig. 1B).

In all study periods, mLCD lowered the BMI compared to the control diet, but it was not statistically significant (Additional file 1: Supplementary Fig. 3A). VLCD significantly decreased the BMI by 1.88 kg/m^2^ (95% CI: − 3.11, − 0.65) for study period of 6 months or less, and 0.82 kg/m^2^ (95% CI: − 1.44, − 0.19) for more than 6 months to 1 year or less compared to the control diet (Additional file 1: Supplementary Fig. 3B).

#### Waist circumference (WC)

mLCD showed a significant decrease in WC (mean difference: -0.65; 95% CI: − 1.16, − 0.14) for the study period of 6 months or less compared to the control diet, but there was no significant difference for a study period of more than 6 months to 1 year or less (36 weeks to 52 weeks), and more than 1 year (Additional file 1: Supplementary Fig. 4A). VLCD showed a decrease in WC compared to the control diet, but there was no statistically significant difference (Additional file 1: Supplementary Fig. 4B).

#### Fat mass and fat percentage

mLCD significantly decreased the fat mass by 0.44 kg (95% CI: − 0.83, − 0.04) for the study period of 6 months or less compared to the control diet, and 0.77 kg (95% CI: − 1.29, − 0.25) for the study period of more than 6 months to 1 year or less. However, there was no significant decrease in a study period of more than 1 year (Additional file 1: Supplementary Fig. 5A). VLCD led to a decreasing trend in the fat mass compared with the control diet in all studies, but it was not statistically significant (Additional file 1: Supplementary Fig. 5B).

In all studies, mLCD did not lead to any significant change in body fat percentage compared with the control diet (Additional file 1: Supplementary Fig. 6A). VLCD resulted to a significant decrease in body fat percentage by 1.88% (95% CI: − 2.87, − 0.89) for 6 months or less compared to the control diet, and 1.56% (95% CI: − 2.41, − 0.71) for more than 6 months to 1 year or less (Additional file 1: Supplementary Fig. 6B).

#### Lipid profile, blood pressure, glycemic control, and other metabolic parameters

Triglycerides (TG), high-density lipoprotein cholesterol (HDL-C), and low-density lipoprotein cholesterol (LDL-C) levels were evaluated. In mLCD, the TG level decreased by 13.76 mg/dl (95% CI: − 19.78, − 7.74) for 6 months or less compared to the control diet (Additional file 1: Supplementary Fig. 7A). In VLCD, the TG level significantly decreased by 21.33 mg/dl (95% CI: − 30.46, − 12.21) for 6 months or less, and 22.52 mg/dl (95% CI: − 31.01, − 14.03) for more than 6 months to 1 year or less (Additional file 1: Supplementary Fig. 7B). mLCD showed a significant increase in HDL-C level compared to control diet in all periods (6 months or less: 2.61 mg/dl [95% CI: 1.34, 3.89], more than 6 months to 1 year or less: 1.45 mg/dl [95% CI: 0.53, 2.37], more than 1 year: 2.84 mg/dl [95% CI: 1.63, 4.05]) (Additional file 1: Supplementary Fig. 7C). Compare to the control diet, VLCD showed an increase in the HDL-C level for the study period of 1 year or less (6 months or less: 4.30 mg/dl [95% CI: 1.79, 6.82], and more than 6 months to 1 year or less: 4.86 mg/dl [95% CI, 2.16, 7.56]) (Additional file 1: Supplementary Fig. 7D). As for LDL-C levels, possible risks were identified, and are discussed further in the “Harms” section (Additional file 1: Supplementary Fig. 7E and 7F).

mLCD reduced the systolic blood pressure (SBP) for all study periods compared to the control diet, but it was not statistically significant (Additional file 1: Supplementary Fig. 8A). For diastolic blood pressure (DBP), there was a statistically significant but minimal decrease only for the study period of more than 6 months to 1 year or less (mean difference: -0.66; 95% CI: − 1.26, − 0.05) (Additional file 1: Supplementary Fig. 8B). VLCD decreased the SBP by 1.97 mmHg (95% CI: − 3.68, − 0.25) for 6 months or less, and 8.1 mmHg (95% CI: − 13.35, − 2.85) for more than 6 months to 1 year or less (Additional file 1: Supplementary Fig. 8C) compared to the control diet, but did not significantly decrease DBP (Additional file 1: Supplementary Fig. 8D).

In mLCD, the fasting blood glucose level was significantly decreased by − 1.62 mg/dl (95% CI: − 2.69, − 0.55) compared to the control diet but only for the study period of more than 6 months to 1 year or less (Additional file 1: Supplementary Fig. 9A). In VLCD, the fasting blood glucose level did not decrease significantly compared to the control diet, irrespective of the study period (Additional file 1: Supplementary Fig. 9B). mLCD significantly decreased the HbA1c level by 0.2% (95% CI: − 0.39, − 0.01) compared to the control diet for the study period of 6 months or less. There was also a decreasing trend over a longer period, but it was not statistically significant (Additional file 1: Supplementary Fig. 9C). VLCD decreased the HbA1c level by 0.27% (95% CI: − 0.50, − 0.03) compared with the control diet for an intervention period of 6 months or less (Additional file 1: Supplementary Fig. 9D). mLCD led to decreasing trends in the fasting insulin levels in all studies compared to the control diet, but it was statistically significant only for 6 months or less (mean difference: -0.94; 95% CI: − 1.73, − 0.16) (Additional file 1: Supplementary Fig. 9E). VLCD decreased the fasting insulin levels in all studies compared to the control diet, but it was not statistically significant (Additional file 1: Supplementary Fig. 9F).

In mLCD, the serum adiponectin level increased in all study periods compared to the control diet, with a significant increase of 0.45 μU/mL (95% CI: 0.15, 0.76) for 6 months or less, and 0.73 μU/mL (95% CI: 0.29, 1.16) for more than 6 months to 1 year or less (Additional file 1: Supplementary Fig. 10A). In VLCD, the serum adiponectin level increased in all study periods compared to the control diet, with a significant increase of 0.75 μg/mL (95% CI: 0.29, 1.21) for 6 months or less in two studies, and 1.30 μg/mL (95% CI: 0.34, 2.26) for more than 1 year in two studies (Additional file 1: Supplementary Fig. 10B).

In terms of CRP levels, mLCD showed a significant decrease of 0.34 mg/L (95% CI: − 0.67, − 0.01) for 6 months or less compared to the control diet (Additional file 1: Supplementary Fig. 10C). VLCD showed a decrease in all studies, but it was not statistically significant (Additional file 1: Supplementary Fig. 10D).

### Summary and conclusion of benefits

In adults with overweight or obesity, the carbohydrate-restricted diets led to weight loss similar to or greater than that of the control diet. As the proportion of carbohydrates decreased, body weight and BMI reduction became greater. When used for 6 months or less, reduction in body weight and BMI was greatest, and the reduction effect decreased as the study period increased. Compared to the control diet, WC is significantly reduced for the study period of 6 months or less in mLCD. Compared to the control diet, fat mass was significantly reduced for the study period of more than 6 months to 1 year or less in mLCD, and body fat percentage was significantly reduced in VLCD. Therefore, a reduction in weight and BMI, as well as WC and body fat mass, can be expected through a short-term carbohydrate-restricted diet for 6 months. Additional effects of TG reduction and HDL-C increase for 1 year or less could also be expected.

### Harms (risks)

#### Fat-free mass

While mLCD led to decreasing trends in fat-free mass in all studies compared to the control diet, it was not statistically significant (Additional file 1: Supplementary Fig. 11A). VLCD significantly decreased the body fat-free mass by 1.05 kg (95% CI: − 1.75, − 0.35) compared to the control diet for a study period of 6 months or less in three studies (Additional file 1: Supplementary Fig. 11B).

#### Lipid profiles and other adverse effects

While mLCD had an increasing trend in LDL-C levels in all studies compared to the control diet, it was not statistically significant (Additional file 1: Supplementary Fig. 7E). VLCD significantly increase the LDL-C by 7.52 mg/dl (95% CI: 3.34, 11.70) for 6 months or less (Additional file 1: Supplementary Fig. 7F).

There was limited literature directly describing the adverse events. In some studies, carbohydrate-restricted diets increase the incidence of nausea, vomiting, headache, and constipation, although it was not statistically significant compared to the control diet in a period of 6 months or less (Additional file 1: Supplementary Fig. 12). Although there is no causal relationship with dietary intervention, two cases of death and one case of coronary artery disease occurred five to 10 months after the beginning of research in the low carbohydrate diet group of the two studies.

### Summary and conclusion of harms

In adults with overweight or obesity, VLCD has been found to cause a decrease in fat-free mass and an increase in LDL-C level compared to the control diet for the study period of 6 months or less. Moreover, caution is required as carbohydrate-restricted diets tend to increase adverse effects such as nausea, vomiting, constipation, and headache during the early period.

### Balance of benefits and risks

In adults with overweight or obesity, carbohydrate-restricted diets led to weight loss similar to or greater than that of the control diet, and the effect was greater as the proportion of carbohydrates decreased. Remarkably, the effect was greatest in a short period of 6 months, and the effect decreased as the period increased. WC is significantly decreased in mLCD compared to the control diet within 6 months, a decrease in fat mass within 1 year, a decrease in TG, and an increase in HDL-C within 6 months. VLCD lowered the fat percentage within 1 year, but an increase in LDL-C, along with a decrease in fat-free mass, within 6 months, requires careful discernment on their application. Moreover, caution is required as carbohydrate-restricted diets tended to increase the incidence of nausea, vomiting, constipation, and headaches during the early period.

### Considerations in the use of the recommendation

1. To reduce body weight in adults with overweight or obesity, a balanced and high-quality diet with carbohydrate restriction and reduced caloric intake is recommended. Recent guidelines for obesity management allow the individualized use of a low carbohydrate diet for obesity treatment [5], and most of the carbohydrate-restricted diets included in this study involved a decrease in total calorie intake.
2. Carbohydrate-restricted diets should reduce total caloric intake while avoiding an increase in saturated and trans-fatty acids intake. In a cohort study examining the association between a carbohydrate-restricted diet and the mortality risk, an animal product-based carbohydrate-restricted diet was associated with increased all-cause mortality both in men and women. In contrast, a vegetable-based carbohydrate-restricted diet was associated with reduced all-cause mortality and cardiovascular mortality [95].
3. The results should be interpreted with caution because the types and amount of carbohydrates, fats, and total calories ingested and the control diet vary across studies. The benefits and risks of the long-term use of these dietary regimens are incompletely understood. In addition, it is necessary to consider the dietary patterns in South Korea, characterized by a much higher rate of carbohydrate intake than in other countries, to improve adherence to these regimens.

## Recommendation and evaluation of evidence for intermittent fasting in adults with overweight or obesity

The recommendation for intermittent fasting (IF) in adults with overweight or obesity will be withheld due to the lack of long-term studies and the heterogeneity of previous studies.

### Level of evidence

In the meta-analysis, ten articles from eight RCTs were included. The analysis included studies conducted with participants classified as overweight or obese, with BMI of ≥23 kg/m^2^, which is the Korean obesity criteria, and most participants in the studies had a mean BMI of ≥30 kg/m^2^. While the risk of bias in individual studies was generally low (Additional file 1: Supplementary Fig. 13), there was a high risk of bias in several items, including indirectness and imprecision. Therefore, the level of evidence for the overall evaluation indicators was evaluated as “very-low evidence” (Additional file 1: Supplementary Table 7).

### Benefits (advantages)

The primary outcomes for assessing the benefit of IF in adults with overweight or obesity included body weight, BMI, WC, fat mass, body fat percentage, and fat-free mass. The secondary outcomes included TG, HDL-C, LDL-C, HbA1c, fasting blood glucose, fasting serum insulin, Homeostatic Model Assessment for Insulin Resistance (HOMA-IR), SBP, and DBP.

#### Body weight, body mass index, and body composition

The intervention group, in whom IF was implemented showed no statistically significant difference in body weight (mean difference: − 1.22 kg; 95% CI: − 3.49, 1.05), BMI (mean difference: − 0.49 kg/m^2^; 95% CI: − 1.13, 0.14), WC (mean difference: − 1.95 cm; 95% CI: − 4.09, 0.20), fat-free mass (mean difference: − 0.35 kg; 95% CI: − 0.87, 0.18), fat mass (mean difference: − 0.67 kg; 95% CI: − 1.95, 0.62), and fat mass percentage (mean difference: 0.27%; 95% CI: − 0.48, 1.01), compared to the control group, within 6 months (12 to 24 weeks). The results of the meta-analysis showed that there was no statistically significant difference in body weight, fat-free mass, and fat mass between the intervention and control group within the 1 year intervention period (Additional file 1: Supplementary Fig. 14).

#### Lipid profile, blood pressure, glycemic control, and other metabolic parameters

As for the secondary outcomes of the meta-analysis of the studies conducted in a period of 12 to 24 weeks, the intervention group showed no statistically significant difference in TG (mean difference: − 1.51 mg/dl; 95% CI: − 17.06, 14.04), HDL-C (mean difference: − 0.17 mg/dl; 95% CI: − 3.27, 2.92), LDL-C (mean difference: − 0.24 mg/dl; 95% CI: − 5.08, 4.59), HbA1c (mean difference: 0.11%; 95% CI: − 0.04, 0.26), fasting blood glucose (mean difference: − 0.89 mg/dl; 95% CI: − 4.30, 2.53), fasting serum insulin (mean difference: − 0.43 μU/mL; 95% CI: − 1.99, 1.14), HOMA-IR (mean difference: − 0.22 mg/dl; 95% CI: − 1.48, 1.05), SBP (mean difference: 0.87 mmHg; 95% CI: − 2.65, 4.39), and DBP (mean difference: − 0.16 mmHg; 95% CI: − 2.89, 2.56) levels, compared to the control group. There was no statistically significant difference in TG, HDL-C, HbA1c, fasting blood glucose, fasting serum insulin, HOMA-IR, SBP, and DBP between the intervention and control group during the 6 month to 1 year intervention period (Additional file 1: Supplementary Fig. 15).

### Harms (risks)

A study reported mild headache and constipation in a patient undergoing alternate-day fasting. Nonetheless, the risks and adverse effects of this intervention were not reported in other studies.

### Balance of benefits and harms

As a result of the IF in adults with overweight or obesity, an increase in LDL-C was observed in the studies conducted for more than 6 months to 1 year or less compared to the control diet. In addition, there were no significant differences in obesity-related outcomes such as body weight, BMI, and WC, as well as glycemic control and lipid profiles. However, an additional analysis of the changes in the intervention group alone revealed that significant reductions in body weight, BMI, WC, body fat mass, and fat-free mass were observed before and after the intervention in all studies. IF for 1 year or less in overweight or obese adults could help reduce body weight similar to the control diet, the continuous calorie restriction diet, or the Dietary Approaches to Stop Hypertension (DASH) diet, but also decrease the fat-free mass, requiring caution.

In the studies included in the analysis, no serious adverse event was observed with IF other than a subtle headache and constipation.

Nevertheless, there are limitations in interpreting and evaluating the overall benefits and risks of IF in adults with overweight or obesity for the following reasons.

1. The studies included in the analysis were very heterogeneous. The studies were conducted in a variety of countries and races, and limited studies were conducted on Asians. Due to the nature of diet research, there were many studies in which blinding was not performed or related information was not described, and the dropout rate of the study also varied from 6 to 31%. Although the analysis was performed under the integrated concept of IF, various heterogeneous dietary regimens such as time-restricted feeding and alternate-day fasting were included. Even within the dietary regimen of the same name, there was a limitation in evaluating the overall effect due to heterogeneity in the period or cycle of fasting. In the studies that conducted alternate day fasting, the intervention was mostly performed with a modified IF method that did not perform complete fasting on the day of intermittent fasting but reduced the intake of calories by about 70–75%. As for the dietary method on the day of intake, there were studies that restricted caloric intake, and there were studies that implemented a standard diet. In two studies, time restricted feeding was implemented as an intervention, and these studies also divided the time of the day and implemented different feeding and fasting times of 16:8 and 12:12.
2. Long-term researches were scarce. The study periods varied from 3 months to a maximum of 1 year, and only one study was conducted for 1 year. Therefore, there is a lack of studies to evaluate the long-term effects of intermittent fasting.

### Considerations in the use of the recommendation

Considering the results of the analysis on benefits and harms as well as the limitations of the above-mentioned studies, recommendation on IF for weight management, diabetes prevention, and cardiovascular risk management in adults with overweight or obesity has been withheld.

## Recommendation and evaluation of evidence for carbohydrate-restricted diets in adults with type 2 diabetes mellitus

### Recommendation 1

In adults with type 2 diabetes, a moderately-low carbohydrate or low carbohydrate diet (mLCD) can be considered a dietary regimen for improving glycemic control and reducing body weight since similar or greater effects on blood glucose-lowering and weight loss are observed compared to generally recommended diets. [Conditional recommendation, moderate quality of evidence].

**Table:** 

**1. A low carbohydrate diet does not imply an extreme reduction in carbohydrate and increase in fat intake, and must not be practiced indiscriminately.** **2. A low carbohydrate diet should reduce caloric intake while avoiding an increase in the intake of saturated and trans fatty acids.**	

### Recommendation 2

In adults with type 2 diabetes, the strong recommendation against a very-low carbohydrate diet (VLCD) as the risks of hypoglycemia and elevated low-density lipoprotein cholesterol (LDL-C) levels outweigh the benefits of blood glucose-lowering and weight loss [Strong recommendation against, moderate quality of evidence].

### Level of evidence

A meta-analysis was performed on the key question, “Are carbohydrate-restricted diets helpful in improving glycemic control in diabetic patients?” Twenty-three articles from 18 RCTs on benefits were included in the analysis. In principle, studies in which more than 50% of all subjects had diabetes were included in the analysis. In 15 studies, 100% of the participants had diabetes [29–31, 45–48, 51, 56, 59, 62, 65, 68, 74–78, 80, 96] and 50% or more had diabetes in two studies [38, 67]. Although less than 50% were diabetic, one study was included in the analysis since the outcomes could be analyzed only for diabetic patients [69, 70]. There were 13 studies on mLCD and five studies on VLCD. Most studies were conducted in Western countries, and no studies were conducted in South Korea. Five studies were conducted in East Asian countries (China, Japan, and Taiwan), with similar demographic characteristics to South Korea. Of 1282 subjects, 369 (28.8%) were from East Asian countries. The risk of bias in individual studies was evaluated as “low” or “some concern” (Additional file 1: Supplementary Fig. 16). However, in evaluating the level of evidence for the key question, the overall dropout rate was high, with heterogeneity in the two groups, with an apparent indirectness. After rating down, the level of evidence for the change in HbA1c level was evaluated as “moderate evidence.” The level of evidence and evaluation results for all variables other than HbA1c can also be seen in Additional file 1: Supplementary Table 8.

### Benefits (advantages)

The primary outcome for assessing the benefit of carbohydrate-restricted diets in diabetic adults for blood glucose control was the change in HbA1c. The secondary outcomes for evaluating the metabolic and cardiovascular benefits of carbohydrate-restricted diets in diabetic adults included body weight, blood pressure, lipid profiles, fasting blood glucose, and HOMA-IR. Table 2 summarizes the meta-analysis results and level of evidence for primary and secondary outcomes.

**Table 2:** Summary of findings for effects of carbohydrate-restricted diets in adults with type 2 diabetes mellitus

Outcome	Illustrative comparative effect^a^ (95% CI)		No. of participants	Quality of the evidence (GRADE)
	Assumed effect (control)	Corresponding effect		
mLCD^b^				
HbA1c, % (follow-up: 8–24 weeks)	−0.2	−0.21 (− 0.32 to − 0.10)	758 (10 studies)	⊕ ⊕ ⊕⊝ Moderate
HOMA-IR (follow-up: 8–24 weeks)	− 0.4	–0.53 (− 0.96 to − 0.11)	248 (3 studies)	⊕ ⊕ ⊝⊝ Low
Fasting glucose, mg/dL (follow-up: 8–24 weeks)	4.65	−9.88 (−18.04 to − 1.71)	337 (6 studies)	⊕ ⊕ ⊝⊝ Low
Body weight, kg (follow-up: 8–24 weeks)	−1.45	−1.54 (−3.11 to 0.02)	619 (8 studies)	⊕ ⊕ ⊝⊝ Low
Systolic blood pressure, mm Hg (follow-up: 8–24 weeks)	−0.25	−2.99 (−5.48 to − 0.49)	510 (6 studies)	⊕ ⊕ ⊕⊝ Moderate
Diastolic blood pressure, mm Hg (follow-up: 8–24 weeks)	0.55	−1.07 (− 2.43 to 0.29)	513 (6 studies)	⊕ ⊕ ⊝⊝ Low
Triglyceride, mg/dL (follow-up: 8–24 weeks)	−4.0	−17.22 (−34.27 to −0.18)	742 (10 studies)	⊕ ⊕ ⊝⊝ Low
LDL-C, mg/dL (follow-up: 8–24 weeks)	−3.6	0.35 (−3.03 to 3.72)	607 (8 studies)	⊕ ⊕ ⊝⊝ Low
HDL-C, mg/dL (follow-up: 8–24 weeks)	0.2	2.30 (0.23 to 4.37)	547 (8 studies)	⊕ ⊕ ⊕⊝ Moderate
Hypoglycemia	There is no study directly evaluated the risk of hypoglycemia. Patients at high risk of hypoglycemia were excluded in 2 out of 13 studies.			
VLCD^c^				
HbA1c, % (follow-up: 12–24 weeks)	−0.2	−0.36 (− 0.54 to − 0.19)	321 (5 studies)	⊕ ⊕ ⊕⊝ Moderate
HOMA-IR (follow-up: 12–24 weeks)	−0.45	−1.07 (−3.13 to 0.98)	119 (2 studies)	⊕ ⊕ ⊝⊝ Low
Fasting glucose, mg/dL (follow-up: 12–24 weeks)	−17.2	−9.64 (− 19.54 to 0.26)	267 (3 studies)	⊕ ⊕ ⊝⊝ Low
Body weight, kg (follow-up: 12–24 weeks)	−3.4	−3.84 (−7.55 to −0.13)	291 (4 studies)	⊕ ⊕ ⊕⊝ Moderate
Systolic blood pressure, mm Hg (follow-up: 12–24 weeks)	−1.7	0.34 (−3.61 to 4.28)	218 (3 studies)	⊕ ⊕ ⊝⊝ Low
Diastolic blood pressure, mm Hg (follow-up: 12–24 weeks)	−2.5	1.38 (−0.90 to 3.67)	218 (3 studies)	⊕ ⊕ ⊝⊝ Low
Triglyceride, mg/dL (follow-up: 12–24 weeks)	−15.7	− 11.40 (− 27.01 to 4.22)	313 (5 studies)	⊕ ⊕ ⊝⊝ Low
LDL-C, mg/dL (follow-up: 12–24 weeks)	− 1.35	7.19 (0.02 to 14.36)	277 (4 studies)	⊕ ⊕ ⊕⊝ Moderate
HDL-C, mg/dL (follow-up: 12–24 weeks)	2.3	0.43 (−1.98 to 2.84)	312 (5 studies)	⊕ ⊕ ⊝⊝ Low
Hypoglycemia	Although no study directly evaluated the risk of hypoglycemia, patients at high risk of hypoglycemia were excluded in 4 out of 5 studies.			
Intermittent fasting^d^				
HbA1c, % (follow-up: 24 weeks)	−0.6	0.10 (−0.35 to 0.55)	63 (1 study)	⊕ ⊕ ⊝⊝ Low
HbA1c, % (follow-up: 52 weeks)	−0.5	0.20 (−0.22 to 0.62)	137 (1 study)	⊕ ⊕ ⊝⊝ Low
Body weight, kg (follow-up: 24 weeks)	−4.0	−1.00 (−6.94 to 4.94)	63 (1 study)	⊕ ⊕ ⊝⊝ Low
Fat-free mass, kg (follow-up: 24 weeks)	−1.1	−1.10 (−2.22 to 0.02)	49 (1 study)	⊕ ⊕ ⊝⊝ Low
Fat mass, kg (follow-up: 24 weeks)	−4.0	0.20 (−1.46 to 1.86)	49 (1 study)	⊕ ⊕ ⊝⊝ Low
Fat mass, % (follow-up: 24 weeks)	−2.1	0.40 (−0.86 to 1.66)	49 (1 study)	⊕ ⊕ ⊝⊝ Low
Hypoglycemia	Although no study directly evaluated the risk of hypoglycemia, most studies in obese or overweight adults have excluded patients with diabetes as an exclusion criterion.			

^a^The basis for the assumed effect is the mean change of outcomes compared to baseline in the control group across studies, and the corresponding effect (and its 95% CI) is based on the assumed effect in the comparison group

^b^mLCD for type 2 diabetes mellitus: Patient or population (patients with type 2 diabetes mellitus), Intervention (mLCD)

^c^VLCD for type 2 diabetes mellitus: Patient or population (patients with type 2 diabetes mellitus), Intervention (VLCD)

^d^Intermittent fasting for type 2 diabetes mellitus: Patient or population (patients with type 2 diabetes mellitus), Intervention (intermittent fasting)

*CI* confidence interval, *GRADE* Grading of Recommendations Assessment, Development and Evaluation, *mLCD* moderately-low carbohydrate or low carbohydrate diet, *HbA1c* glycosylated hemoglobin, *HOMA-IR* homeostatic model assessment for insulin resistance, *LDL-C* low-density lipoprotein cholesterol, HDL-C high-density lipoprotein cholesterol, *VLCD* very-low carbohydrate diet

GRADE Working Group grades of evidence: High quality (Further research is very unlikely to change our confidence in the estimate of effect); Moderate quality (Further research is likely to have an important impact on our confidence in the estimate of effect and may change the estimate); Low quality (Further research is very likely to have an important impact on our confidence in the estimate of effect and is likely to change the estimate); Very low quality (We are very uncertain about the estimate).

#### HbA1 reduction

**Fig. 2.**
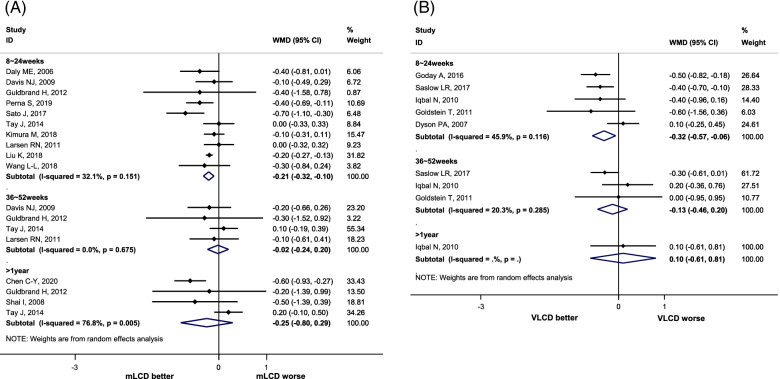
Effect of Carbohydrate-Restricted Diets on Glycated Hemoglobin (HbA1c) in Adults with Type 2 Diabetes. **A** Moderately-low carbohydrate or low carbohydrate diets (mLCD). **B** Very-low carbohydrate diets (VLCD)

Compared to the control diets, mLCD (12 studies) and VLCD (five studies) reduced the HbA1c levels by 0.21% (95% CI -0.32, − 0.10) and 0.36% (95% CI: − 0.54, − 0.19) within 6 months, respectively. However, no additional benefit was observed for periods exceeding 6 months (Fig. 2). In addition, the results of five studies conducted in East Asia also showed similar results (within 6 months, mean difference: -0.26; 95% CI -0.44, − 0.07) (Additional file 1: Supplementary Fig. 17). Compared to before the intervention, mLCD lowered the HbA1c by 0.50% (95% CI -0.63, − 0.37), and VLCD lowered the HbA1c by 0.60% (95% CI -1.12, − 0.08) within 6 months after the intervention. Therefore, both mLCD and VLCD additionally improved glycemic control within 6 months in adults with type 2 diabetes compared to the control diet with an equivalent reduction in caloric intake.

#### Body weight, blood pressure, lipid profiles, fasting blood glucose, and insulin resistance

In body weight, mLCD (11 studies) reduced by 1.54 kg (95% CI -3.11, 0.02) within 6 months compared to the control diets, although it was not statistically significant. A weight loss of 3.84 kg (95% CI -7.55, − 0.13) was also observed in VLCD (five studies). However, no additional benefit was observed for periods exceeding six months. (Additional file 1: Supplementary Fig. 18). These benefits were consistent with the meta-analysis results, including more studies in obese adults. Hence, mLCD and VLCD elicit additional weight loss within 6 months in diabetic patients compared to the control diet.

In blood pressure, mLCD (seven studies) reduced the SBP by 2.99 mmHg (95% CI -5.48, − 0.49) was observed within 6 months compared to the control diets, but no such benefit was observed with VLCD (three studies). For DBP, both diets did not show any blood pressure-lowering effect in any period (Additional file 1: Supplementary Fig. 19). These results were inconsistent with the meta-analysis results, including more studies in obese adults. Hence, the additional blood pressure-lowering effect of mLCD and VLCD compared to control diets in diabetic patients is inconclusive.

In lipid profiles, TG, HDL-C, and LDL-C levels were evaluated as outcomes. Compared to the control diets, mLCD (11 studies) decrease in TG level by 17.22 mg/dl (95% CI -34.27, − 0.18) within 6 months, and 17.85 mg/dl (95% CI -32.08, − 3.62) for 1 year or less. It also resulted to an additional increase in HDL-C level of 2.30 mg/dl (95% CI 0.23, 4.37) for 6 months or less, and 1.77 mg/dl (95% CI 0.01, 3.53) for 1 year or less. However, for LDL-C levels, no additional reduction was confirmed in all periods. In VLCD, no benefits in TG and HDL-C levels were observed (five studies). Rather, an additional increase in LDL-C level of 7.19 mg/dl (95% CI 0.02, 14.36) was observed within 6 months (Additional file 1: Supplementary Fig. 20). These findings were consistent with the meta-analysis results, including more studies in obese adults. Hence, even in diabetic patients, mLCD has additional benefits of improving TG and HDL-C levels within 1 year, whereas VLCD has a risk of increasing LDL-C levels within 6 months compared to the control diets.

In fasting blood glucose, mLCD (seven studies) caused an additional reduction of 9.88 mg/dl (95% CI -18.04, − 1.71) within 6 months compared to the control diet. Although not statistically significant, VLCD (three studies) also reduced blood glucose level by 9.64 mg/dl (95% CI -19.54, 0.26). However, no additional benefits were observed for periods exceeding 6 months. (Additional file 1: Supplementary Fig. 21). These findings were consistent with the meta-analysis results, including more studies in obese adults. Hence, mLCD and VLCD additionally improved fasting blood glucose levels within 6 months compared to the control diets.

HOMA-IR was examined as an outcome for the evaluation of insulin resistance. In the five studies included in the HOMA-IR analysis, mLCD (3 studies) led to an additional reduction of 0.53 (95% CI -0.96, − 0.11) within 6 months compared to the control diets. At the same time, VLCD (two studies) reduced HOMA-IR by 1.07 (95% CI -3.13, 0.98), although it was not statistically significant (Additional file 1: Supplementary Fig. 22). Hence, mLCD additionally improved insulin resistance within 6 months compared to the control diets.

### Summary and conclusion of benefits

In adults with type 2 diabetes, mLCD and VLCD were more effective in reducing body weight and improving glycemic control than equivalent calorie-restricted diets within 6 months. The benefits were greater in VLCD than in mLCD. In addition, mLCD improved insulin resistance and lipid profile (decreased TG and increased HLD-C levels).

### Harms (risks)

Of the 18 studies, eight studies mentioned risks, and five of them stated that there were “no serious adverse events.” Three studies reported adverse events, but they were evaluated to not be associated with the intervention.

Gastrointestinal disturbance, such as nausea, vomiting, and constipation, tended to increase within a short period but decreased afterward in two VLCD studies for the risk assessment in obese adults (Additional file 1: Supplementary Fig. 12) [17, 45].

Hypoglycemia is the most concerning complication of reducing total calorie intake or carbohydrate-restricted diets in diabetic patients. There was no direct evidence that carbohydrate-restricted diets were more likely to cause hypoglycemia than a calorie-restricted diet. However, patients with type 1 diabetes or a history of severe hypoglycemia, or patients using insulin or sulfonylureas at a higher risk of hypoglycemia were also excluded in several studies [38, 45, 51, 59, 67, 74–78]. Furthermore, it is clear that the risk of hypoglycemia increase with a greater degree of carbohydrate or calorie restriction [96]. Indeed, only 2 of 13 mLCD studies (15.4%) excluded patients at a higher risk of hypoglycemia with type 1 diabetes or those using insulin or sulfonylureas [59, 74–78], compared to 4 of 5 VLCD studies (75.0%) [38, 45, 51, 67] in our analysis. Therefore, carbohydrate- or calorie-restricted diets may increase the risk of hypoglycemia, which was greater in VLCD.

LDL-C level was statistically significantly increased by VLCD compared to the control diet in the meta-analysis results. These results were consistent with the meta-analysis results, including more studies in obese adults. Hence, VLCD is likely to increase the risk of LDL-C.

### Summary and conclusion of harms

Many studies excluded patients with a higher risk of hypoglycemia, and this risk is higher as the degree of carbohydrate restriction increases [97]. Therefore, VLCD is associated with the highest risk of hypoglycemia. LDL-C levels, an important risk factor for cardiovascular disease, increased significantly in VLCD but not in mLCD. Diabetes is a significant contributor to cardiovascular disease, and the prevalence of cardiovascular disease is two to four times higher in patients with diabetes than in those without diabetes [98]. Therefore, elevated LDL-C in VLCD is a potential risk factor for cardiovascular disease.

### Balance of benefits and harms

In adults with type 2 diabetes, carbohydrate-restricted diets improved glycemic control and reduced body weight to a level similar to or greater than that of the control diets, and the effects were greater as carbohydrate intake decreased. Moreover, the effects were greatest within 6 months and decreased as the intervention period increased. mLCD decreased TG, increased HDL-C. VLCD elevated LDL-C and significantly increased the risk of hypoglycemia. Therefore, the benefits outweigh the risks in mLCD but not in VLCD.

### Considerations in the use of the recommendation

Participants with malignancies or serious medical conditions in the cardiovascular system, kidney, gastrointestinal tract, and pancreas; pregnant or lactating women; psychiatric disorders including eating disorders or drug abuse; acute illnesses such as infections, were excluded due to the greater risk of harm in most studies, because carbohydrate-restricted diets have a high potential for harm in such patients and inconclusive benefits. Therefore, it is inappropriate to recommend a carbohydrate-restricted diet to those patients.

Recently popular dietary approaches, such as extremely low carbohydrate and high-fat diets, focus only on lowering the carbohydrate ratio, neglecting the total caloric intake and fat quality. Since reducing carbohydrate intake inevitably leads to an increase in fat intake, we should also be concerned about total fat intake and fat quality. Fortunately, the researchers in most studies tried to minimize the increase in fat intake while reducing the total caloric intake included in the analysis. Simultaneously, they attempted to decrease the intake of saturated and trans-fatty acids, with potential harm to cardiovascular disease. Therefore, carbohydrate-restricted diets should reduce total caloric intake while avoiding an increase in the intake of saturated and trans fatty acids.

Since most studies are focused on evaluating the benefits of carbohydrate-restricted diets, with a scarcity of research on risk assessment, further studies are required. Furthermore, well-designed RCTs in Korean patients are also needed. Considering that the carbohydrate intake rate of Koreans is about 65%, which is significantly higher than that of other countries [93], future studies must evaluate 1) whether this benefit can be maintained even after adjusting the carbohydrate restriction rate to 45 ~ 55%, which is higher than that of MCD, and 2) whether carbohydrate restriction to the level of MCD or LCD would be harmful to Koreans.

## Recommendation and evaluation of evidence for intermittent fasting in adults with type 2 diabetes mellitus

### Recommendation

In adults with type 2 diabetes, strong recommendation against intermittent fasting (IF) due to the lack of evidence on its benefits and harms, and risk of hypoglycemia [Strong recommendation against, low quality of evidence].

### Level of evidence

Eight randomized controlled trials in 10 articles were conducted for evaluating the benefits of IF in overweight and obese adults, but only one study was conducted with type 2 diabetes patients. Table 2 shows the results in 2 literature of 1 research [85, 86] and the assessment of the level of evidence performed in diabetic patients. The risk of bias in the study was evaluated as “low” or “some concern” (Additional file 1: Supplementary Fig. 13). However, there was only one study to be analyzed, the number of subjects was too small, and the effect of outcome was insufficient, with apparent imprecision. After rating down, the level of evidence for the change in HbA1c level was evaluated as “low evidence”.

### Benefits (advantages)

No additional benefit was identified in one study on patients with type 2 diabetes compared to a control diet.

### Harms (risks)

The single study conducted on patients with type 2 diabetes had no description of the risks. In other studies, diabetes itself is often used as an exclusion criterion of participants due to the associated risks, including hypoglycemia, in diabetic patients during the fasting period.

### Balance of benefits and harms

In adults with type 2 diabetes, IF has no evidence of benefit for metabolic outcomes such as glycemic control and weight loss, and no evidence of harm has been identified. However, there is a high risk of harm such as hypoglycemia during the fasting period. In conclusion, this committee decided that IF was a “strong recommendation against” in adults with type 2 diabetes mellitus.

### Considerations in the use of the recommendation

Additional research is needed to establish sufficient evidence, considering the continuing and high interest in IF as an attractive diet regimen among the general population with some studies publishing beneficial results. Although few studies have been conducted in diabetic patients due to hypoglycemia, research on these patients can be possible with the widespread use of various antidiabetic drugs with a low risk of hypoglycemia.

## Recommendation and evaluation of evidence for carbohydrate-restricted diets and intermittent fasting in adults with hypertension

1. As there is insufficient evidence to support carbohydrate-restricted diets in adults with hypertension, it has been decided not to present a recommendation.
2. As there is insufficient evidence to support intermittent fasting (IF) in adults with hypertension, it has been decided not to present a recommendation.

### Level of evidence

#### Carbohydrate-restricted diets

The inclusion criteria were studies conducted on patients with hypertension; studies in which ≥50% of participants were diagnosed with hypertension; studies in which ≥50% of participants were taking antihypertensive drugs; and studies with patients having SBP of 140 mmHg or higher, or DBP of 90 mmHg or higher at baseline. In the meta-analysis, 14 articles from six RCTs on benefit were included. There were three studies each on both mLCD and VLCD. Each study was evaluated to be designed with low or some concerns (Additional file 1: Supplementary Fig. 23). In the evaluation of the level of evidence with respect to the key question, however, there was a high risk of bias in several items, including indirectness and imprecision with no studies targeting only patients with hypertension, with the absence of blinding in most of the studies due to the nature of the research, high dropout rates, and heterogeneity between groups. Therefore, the level of evidence was downgraded to “very-low evidence” (Additional file 1: Supplementary Table 9).

#### Intermittent fasting

There have been no studies on the benefits of IF conducted on the included studies. Therefore, the evidence for IF was not evaluated.

### Benefits (advantages)

Changes in SBP and DBP were the primary outcomes to determine the benefits of carbohydrate-restricted diets in adults with hypertension. The secondary outcomes for assessing the metabolic and cardiovascular benefits of carbohydrate-restricted diets included body weight, lipid profiles of cholesterol and triglycerides. Table 3 summarizes the level of evidence and meta-analysis results for primary and secondary outcomes.

**Table 3:** Summary of findings for effects of carbohydrate-restricted diet in adults with hypertension

Outcome	Illustrative comparative effect^a^ (95% CI)		No. of participants	Quality of the evidence (GRADE)
	Assumed effect (control)	Corresponding effect		
mLCD^b^				
Systolic blood pressure, mm Hg (follow-up: 8–24 weeks)	−4.55	−3.25 (−7.28 to 0.77)	195 (2 studies)	⊕⊝⊝⊝ Very Low
Diastolic blood pressure, mm Hg (follow-up: 8–24 weeks)	−4.00	−1.80 (− 4.56 to 0.96)	93 (1 study)	⊕⊝⊝⊝ Very Low
Triglyceride, mg/dL (follow-up: 8–24 weeks)	−15.48	−35.58 (−52.84 to − 18.33)	195 (2 studies)	⊕⊝⊝⊝ Very Low
LDL-C, mg/dL (follow-up: 8–24 weeks)	−0.30	0.00 (−9.55 to 9.55)	93 (1 study)	⊕⊝⊝⊝ Very Low
HDL-C, mg/dL (follow-up: 36–52 weeks)	2.3	1.60 (−1.13 to 4.33)	93 (1 study)	⊕⊝⊝⊝ Very Low
Body weight, kg (follow-up: 8–24 weeks)	−6.2	−1.81 (−3.93 to 0.30)	195 (2 studies)	⊕⊝⊝⊝ Very Low
FMD, % (follow-up: 36–52 weeks)	−0.6	0.30 (− 0.58 to 1.18)	93 (1 study)	⊕⊝⊝⊝ Very Low
VLCD^c^				
Systolic blood pressure, mm Hg (follow-up: 8–24 weeks)	−6.3	−1.34 (−5.20 to 2.51)	232 (2 studies)	⊕⊝⊝⊝ Very Low
Diastolic blood pressure, mm Hg (follow-up: 8–24 weeks)	−4.0	2.01 (−0.61 to 4.63)	232 (2 studies)	⊕⊝⊝⊝ Very Low
Triglyceride, mg/dL (follow-up: 8–24 weeks)	−19.95	−10.17 (−43.00 to 22.67)	232 (2 studies)	⊕⊝⊝⊝ Very Low
LDL-C, mg/dL (follow-up: 8–24 weeks)	−6.75	8.91 (−9.27 to 27.08)	232 (2 studies)	⊕⊝⊝⊝ Very Low
HDL-C, mg/dL (follow-up: 8–24 weeks)	2.75	1.85 (−5.98 to 9.69)	232 (2 studies)	⊕⊝⊝⊝ Very Low
Body weight, kg (follow-up: 8–24 weeks)	−6.05	−1.16 (−2.65 to 0.34)	232 (2 studies)	⊕⊝⊝⊝ Very Low
FMD, % (follow-up: 36–52 weeks)	−0.3	−1.80 (−3.48, − 0.12)	49 (1 study)	⊕⊝⊝⊝ Very Low

^a^The basis for the assumed effect is the mean change of outcomes compared to baseline in the control group across studies, and the corresponding effect (and its 95% CI) is based on the assumed effect in the comparison group

^b^mLCD for hypertension: Patient or population (patients with hypertension), Intervention (mLCD)

^c^VLCD for hypertension: Patient or population (patients with hypertension), Intervention (VLCD)

*CI* confidence interval, *GRADE* Grading of Recommendations Assessment, Development and Evaluation, *mLCD* moderately-low carbohydrate or low carbohydrate diet, *LDL-C* low-density lipoprotein cholesterol, *HDL-C* high-density lipoprotein cholesterol, *FMD* flow-mediated dilatation, *VLCD* very-low carbohydrate diet

GRADE Working Group grades of evidence: High quality (Further research is very unlikely to change our confidence in the estimate of effect); Moderate quality (Further research is likely to have an important impact on our confidence in the estimate of effect and may change the estimate); Low quality (Further research is very likely to have an important impact on our confidence in the estimate of effect and is likely to change the estimate); Very low quality (We are very uncertain about the estimate).

### Blood pressure control

**Fig. 3.**
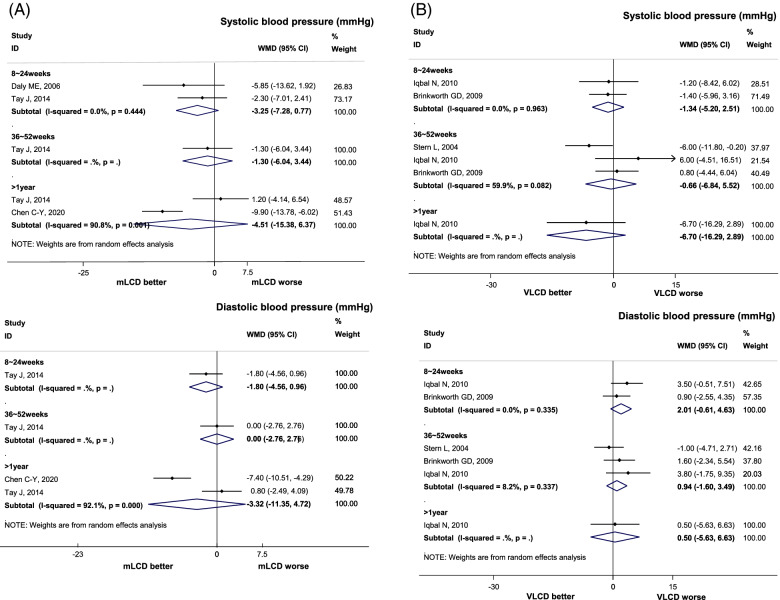
Effect of Carbohydrate-Restricted Diets on Systolic and Diastolic Blood Pressure in Adults with Hypertension. **A** Moderately-low carbohydrate or low carbohydrate diets (mLCD). **B** Very-low carbohydrate diets (VLCD)

Compared to the control diet, mLCD significantly decreased SBP in 8 to 24 weeks and marginally decreased SBP in intervention periods longer than 36 weeks (Fig. 3A). mLCD did not significantly affect DBP (Fig. 3A). However, with mLCD, there was a significant reduction in BP levels compared to the baseline within 1 year (SBP, 6 months or less: mean difference − 9.28, 95% CI -13.76, − 4.80, more than 6 months to 1 year or less: mean difference − 7.10, 95% CI -10.37, − 3.83, more than 1 year: mean difference − 5.27, 95% CI -11.44, 0.90; DBP, 6 months or less: mean difference − 8.20, 95% CI -9.82, − 6.58, more than 6 months to 1 year or less: mean difference − 6.20, 95% CI -8.16, − 4.24, more than 1 year: mean difference − 3.14, 95% CI -6.86, 0.58). The absence of a difference between the intervention group and the control group did not necessarily mean that the low carbohydrate diet did not have a benefit associated with blood pressure. VLCD marginally decreased SBP compared with the control diet (Fig. 3B). VLCD did not lead to a significant difference in DBP compared to the control diet (Fig. 3B).

#### Body weight, lipid profile, fasting blood glucose, and insulin resistance

In terms of body weight, mLCD and VLCD did not show an additional reduction effect than the control diet (Additional file 1: Supplementary Fig. 24). mLCD significantly decreased TG levels compared with the control diet; however, the significant difference between the two groups disappeared in intervention periods longer than 1 year. mLCD significantly increased the HDL-C levels by 3.36 mg/dl (95% CI: 0.71, 6.00) after 1 year relative to the control diet. VLCD did not significantly change the body weight and TG compared to the control diet. However, there is a significant increase in HDL-C level by 4.11 mg/dl (95% CI: 0.81, 7.42) for more than 6 months to 1 year or less, which disappeared after 1 year (Additional file 1: Supplementary Fig. 25).

### Harms (risks)

mLCD marginally increased LDL-C levels, whereas VLCD significantly increased LDL-C in an intervention period of 6 months to 1 year compared with the control diet (Additional file 1: Supplementary Fig. 25). High participant dropout was observed during the study period, suggesting difficulty in maintaining carbohydrate-restricted diets. In addition, although rare, deaths have been reported in the VLCD group during the observation period in some studies. During the 24-month observation period, one patient died from myocardial infarction in both the intervention and control groups. During the 12-month observation period, there was one death due to ischemic cardiomyopathy and one death due to hyperosmolar coma in the intervention group, and one patient was hospitalized with noncardiac chest pain. Although it has not been proven that each death is directly related to a VLCD, further assessment of the risk will be needed. In some low carbohydrate diet studies, 14.0% of musculoskeletal disorders occurred in the low carbohydrate intervention group during the observation period, and 22.4% of musculoskeletal disorders occurred in the control group. Although rare, during the 52-week observation period, one patient in the intervention group was hospitalized for arrhythmia suspected of heart failure, and one patient underwent a hypoglycemic event without being hospitalized. Although it has not been proven that each event is directly caused by a low carbohydrate diet, further assessment of the risk is needed.

### Balance of benefits and risks

For obese adults with hypertension, carbohydrate-restricted diets had no additional benefit in reducing blood pressure and weight compared to calorie-restricted or low fat diets when maintained for 1 year or longer. While TG levels decreased within 1 year, such a benefit reduced after 1 year. There was a risk of increasing LDL-C for less than 1 year in VLCD, but thereafter, the risk disappeared. Although it has not been proven as a direct result of carbohydrate-restricted diets, cardiovascular death and hospitalization have been reported. Therefore, the selection of such methods should be made by fully considering the potential benefits and risks according to the condition of each patient. Accordingly, the benefits and risks remain unclear. Additional research results in patients with hypertension will be needed in the future.

### Considerations in the use of the recommendation

Further research is needed to establish the evidence, as there is insufficient evidence to prove the effectiveness of LCD in adults with hypertension, and there are no randomized controlled trials targeting only patients with hypertension, with no studies presenting the change in blood pressure as a primary outcome.

## Conclusions

Our committee decided that mLCD is a conditional recommendation as a dietary regimen for reducing body weight in adults with overweight or obesity and for improving glycemic control and reducing body weight in adults with type 2 diabetes. In contrast, VLCD and IF are recommended to avoid for adults with type 2 diabetes.

## Supplementary Information

**Additional file 1. Supplementary Table 1.** Search Strategy in MEDLINE through PubMed. **Supplementary Table 2.** The framework of PICO in developing the focused question. **Supplementary Table 3.** Characteristics of randomized controlled trials included in the meta-analysis to evaluate the effects of carbohydrate-restricted diets. **Supplementary Table 4.** Characteristics of randomized controlled trials included in the meta-analysis to evaluate the effects of intermittent fasting. **Supplementary Table 5.** Classification of carbohydrate-restricted diets. **Supplementary Table 6.** Quality of the evidence assessment for included studies evaluating the effects of carbohydrate-restricted diets in adults with overweight/obesity. **Supplementary Table 7.** Quality of the evidence assessment for included studies evaluating the effects of intermittent fasting (IF) in adults with overweight/obesity. **Supplementary Table 8.** Quality of the evidence assessment for included studies evaluating the effects of carbohydrate-restricted diets in adults with diabetes. **Supplementary Table 9.** Quality of the evidence assessment for included studies evaluating the effects of carbohydrate-restricted diets in adults with hypertension. **Supplementary Fig. 1.** PRISMA study flow for literature selection and exclusion process. **Supplementary Fig. 2.** Risk of bias assessment in studies evaluating the effects of carbohydrate-restricted diets in adults with overweight/obesity. **Supplementary Fig. 3.** Effects of carbohydrate-restricted diet on body mass index (BMI) in adults with overweight/obesity. **Supplementary Fig. 4.** Effects of carbohydrate-restricted diets on waist circumference in adults with overweight/obesity. **Supplementary Fig. 5.** Effects of carbohydrate-restricted diets on fat mass in adults with overweight/obesity. **Supplementary Fig. 6.** Effects of carbohydrate-restricted diets on body fat percentage in adults with overweight/obesity. **Supplementary Fig. 7.** Effects of carbohydrate-restricted diets on serum lipid profile in adults with overweight/obesity. **Supplementary Fig. 8.** Effects of carbohydrate-restricted diets on blood pressure in adults with overweight/obesity. **Supplementary Fig. 9.** Effects of carbohydrate-restricted diets on fasting glucose, HbA1c, and fasting insulin levels in adults with overweight/obesity. **Supplementary Fig. 10.** Effects of carbohydrate-restricted diets on serum adiponectin and C reactive protein (CRP) levels in adults with overweight/obesity. **Supplementary Fig. 11.** Effects of carbohydrate-restricted diets on fat free mass in adults with overweight/obesity. **Supplementary Fig. 12.** Adverse events reported regarding carbohydrate-restricted diets in adults with overweight/obesity. **Supplementary Fig. 13.** Risk of bias assessment in studies evaluating the effects of intermittent fasting in adults with overweight/obesity. **Supplementary Fig. 14.** Effect of Intermittent fasting on (A) Body weight, (B) Body mass index, (C) Waist circumference, (D) Fat free mass, (E) Fat mass, and (F) Fat mass in adults with overweight/obesity. **Supplementary Fig. 15.** Effect of Intermittent fasting on (A) Triglyceride (mg/dl), (B) HDL cholesterol (HDL-C, mg/dl), (C) LDL cholesterol (LDL-C, mg/dl), (D) Hemoglobin A1C (%), (E) Fasting glucose (mg/dl), (F) Fasting insulin (μU/mL), (G) HOMA-IR (%), (H) Systolic blood pressure (mmHg), and (I) Diastolic blood pressure(mmHg) in adults with overweight/obesity. **Supplementary Fig. 16.** Risk of bias assessment in studies evaluating the effects of carbohydrate-restricted diets in adults with diabetes. **Supplementary Fig. 17.** Effect of carbohydrate-restricted diets on glycosylated hemoglobin (HbA1c) in adults with diabetes in East-Asian countries (China, Japan, Taiwan). **Supplementary Fig. 18.** Effect of carbohydrate-restricted diets on body weight in adults with diabetes. **Supplementary Fig. 19.** Effect of carbohydrate-restricted diets on blood pressure in adults with diabetes. **Supplementary Fig. 20.** Effect of carbohydrate-restricted diets on lipid profiles in adults with diabetes. **Supplementary Fig. 21.** Effect of carbohydrate-restricted diets on fasting glucose in adults with diabetes. **Supplementary Fig. 22.** Effect of carbohydrate-restricted diets on insulin resistance (HOMA-IR) in adults with diabetes. **Supplementary Fig. 23.** Risk of bias assessment in studies evaluating the effects of carbohydrate-restricted diets in adults with hypertension. **Supplementary Fig. 24.** Effect of carbohydrate-restricted diets on body weight in adults with hypertension. **Supplementary Fig. 25.** Effect of carbohydrate-restricted diets on lipid profile in adults with hypertension

## Data Availability

The datasets used and/or analyzed during the current study are available from the corresponding author on reasonable request.
