# Supplementary material for: Effect of carbohydrate-restricted diets and intermittent fasting on obesity, type 2 diabetes mellitus, and hypertension management: consensus statement of the Korean Society for the Study of obesity, Korean Diabetes Association, and Korean Society of Hypertension
Source: Clin Hypertens. 2022 Jun 1;28:26. doi: 10.1186/s40885-022-00207-4 (PMC9158277; doi:10.1186/s40885-022-00207-4)
Supplement: Supplementary file 1 — Additional file 1. Supplementary Table 1. Search Strategy in MEDLINE through PubMed. Supplementary Table 2. The framework of PICO in developing the focused question. Supplementary Table 3. Characteristics of randomized controlled trials included in the meta-analysis to evaluate the effects of carbohydrate-restricted diets. Supplementary Table 4. Characteristics of randomized controlled trials included in the meta-analysis to evaluate the effects of intermittent fasting. Supplementary Table 5. Classification of carbohydrate-restricted diets. Supplementary Table 6. Quality of the evidence assessment for included studies evaluating the effects of carbohydrate-restricted diets in adults with overweight/obesity. Supplementary Table 7. Quality of the evidence assessment for included studies evaluating the effects of intermittent fasting (IF) in adults with overweight/obesity. Supplementary Table 8. Quality of the evidence assessment for included studies evaluating the effects of carbohydrate-restricted diets in adults with diabetes. Supplementary Table 9. Quality of the evidence assessment for included studies evaluating the effects of carbohydrate-restricted diets in adults with hypertension. Supplementary Fig. 1. PRISMA study flow for literature selection and exclusion process. Supplementary Fig. 2. Risk of bias assessment in studies evaluating the effects of carbohydrate-restricted diets in adults with overweight/obesity. Supplementary Fig. 3. Effects of carbohydrate-restricted diet on body mass index (BMI) in adults with overweight/obesity. Supplementary Fig. 4. Effects of carbohydrate-restricted diets on waist circumference in adults with overweight/obesity. Supplementary Fig. 5. Effects of carbohydrate-restricted diets on fat mass in adults with overweight/obesity. Supplementary Fig. 6. Effects of carbohydrate-restricted diets on body fat percentage in adults with overweight/obesity. Supplementary Fig. 7. Effects of carbohydrate-restricted diets on serum lipid profi [file 40885_2022_207_MOESM1_ESM.docx]

**Supplementary Table 1.** Search strategy in Medline through PubMed

| 1. "Diet, Carbohydrate-Restricted"[Mesh] 2. (Diet[TIAB] OR Diets[TIAB] OR Dietary[TIAB]) AND ((carbohydrate[TIAB] OR Carbohydrates[TIAB]) AND (Restricted[TIAB] OR Low[TIAB] OR restriction[TIAB] OR restrictive[TIAB] OR Reducing[TIAB] OR Reduction[TIAB] OR Modification[TIAB] OR Modifications[TIAB])) 3. (Diet[TIAB] OR Diets[TIAB]) AND Ketogenic[TIAB] 4. (Intermittent[TIAB] OR "time restricted"[TIAB] OR Alternate[TIAB] OR periodic[TIAB] OR ramadan[TIAB]) AND (Fasting[TIAB] OR energy[TIAB] OR feeding[TIAB] OR diet[TIAB] OR diets[TIAB] OR meal[TIAB] OR eating[TIAB] OR caloric[TIAB] OR calorie[TIAB]) 5. "meal skipping"[TIAB] OR "meal frequency"[TIAB] 6. 1–5/OR 7. 6 AND (groups[tiab] OR trial[TIAB] OR randomly[TIAB] OR placebo[TIAB] OR randomized[TIAB] OR "controlled clinical trial"[PT] OR "randomized controlled trial"[PT]) NOT (animals[MH] NOT (humans[MH] AND animals[MH])) 8. 7 AND Filters applied: from 2000/1/1 – 2021/6/8 9. 8 NOT "review"[Publication Type] OR "review literature as topic"[MeSH Terms] 10. 9 NOT ((meta-analysis[Filter] OR systematic review[Filter]) |
| --- |

**Supplementary Table 2.** The framework of PICO in developing the focused question

| PICO | Description of detail |
| --- | --- |
| Population (P) | • Obese or overweight adults (BMI ≥23 kg/m^2^) or  • Patients with T2DM or  • Patients with hypertension |
| Intervention (I) | • PICO1: carbohydrate-restricted diets: moderately-low, low, very-low carbohydrate diet or  • PICO2: intermittent fasting: time-restriction eating, alternate-day fasting, intermittent energy restriction |
| Comparator (C) | Isocaloric diets: standard, calorie-restricted, low-fat, Mediterranean, DASH diets, etc. |
| Outcome (O) | Body weight, BMI, WC, fat mass, fat-free mass, blood pressure, lipid profile (TC, LDL-C, HDL-C, TG), HbA1c, fasting plasma glucose, fasting plasma insulin, HOMA-IR, CRP, adiponectin, FMD, adverse effects, etc. |
| Study design (S) | Randomized controlled clinical trials that conducted more than 8 weeks |
| Target | • Obese or overweight adults or  • Patients with T2DM or  • Patients with hypertension  • Healthcare professionals who manage patients with obesity, T2DM, or hypertension |

PICO, population, intervention, comparator, outcome; BMI, body mass index; T2DM, type 2 diabetes mellitus; DASH, dietary approaches to stop hypertension; WC, waist circumference; TC, total cholesterol; LDL-C, low-density lipoprotein cholesterol; HDL-C, high-density lipoprotein cholesterol; TG, triglyceride; HbA1c, glycosylated hemoglobin; HOMA-IR, homeostatic model assessment for insulin resistance; CRP, C-reactive protein; FMD, flow-mediated dilation.

**Supplementary Table 3.** Characteristics of randomized controlled trials included in the meta-analysis to evaluate the effects of carbohydrate-restricted diets

| Study | Population | Nationality | Duration of study | Control | Intervention | Follow-up loss rate (control:intervention), % | Subject number | Asian, % | FPG, mg/dL | TC, mg/dL | TG, mg/dL | LDL-C, mg/dL | HDL-C, mg/dL | BW, kg | BMI, kg/m^2^ | HbA1c, % | SBP, mm Hg | DBP, mm Hg |
| --- | --- | --- | --- | --- | --- | --- | --- | --- | --- | --- | --- | --- | --- | --- | --- | --- | --- | --- |
| Aronica et al. (2021) [16] | Ob | US | 1 yr | LFD | LCD | 21.0:21.1 | 609 | 9.9 | 98.6 | NA | 128.4 | 112.9 | 49.9 | 96.9 | 33.4 | NA | 122.9 | 81.1 |
| Bazzano et al. (2014) [17,18] | Ob | US | 1 yr | LFD | VLCD | 17.8:21.3 | 148 | 0.7 | 94.0 | 201.5 | 119.0 | 122.6 | 55.1 | 97.1 | 35.4 | NA | 122.6 | 78.4 |
| Bhattacharyya et al. (2012) [19] | Ob | India | 6 mo | SD | VLCD | NA | 150 | NA | NA | NA | NA | NA | NA | NA | 27.1 | NA | NA | NA |
| Brinkworth et al. (2004) [20] | Ob | Australia | 1 yr | SD | MCD | 24.1:27.6 | 43 | NA | 96.3 | NA | NA | NA | NA | 94.0 | 34.1 | NA | NA | NA |
| Brinkworth et al. (2009) [21], Brinkworth et al. (2010) [22], Brinkworth et al. (2011) [23], Wycherley et al. (2011) [24], Tay et al. (2008) [25], Wycherley et al. (2010) [26] | Ob, HTN | Australia | 1 yr | LFD | VLCD | 40.9:42.1 | 118 | NA | 101.8 | 210.8 | 153.9 | 127.7 | 54.3 | 95.3 | 33.7 | NA | 134.0 | 74.8 |
| Cardillo et al. (2006) [27] | Ob | US | 3 yr | LFD | VLCD | NA | 53 | NA | 116.5 | NA | NA | NA | NA | NA | 43.3 | NA | NA | NA |
| Zinn et al. (2017) [28] | Ob | New Zealand | 3 mo | LFD | MCD | 40:33 | 41 | 0 | 92.7 | NA | 128.4 | 112.1 | 44.5 | 91.8 | 31.1 | 5.4 | NA | NA |
| Chen et al. (2020) [29] | Ob, T2DM, HTN | Taiwan | 18 mo | SD | LCD | 6.7:8.5 | 85 | 100 | 160.3 | 177.6 | 170.7 | 103.4 | 45.4 | 69.0 | 26.9 | 8.6 | 130.4 | 75.4 |
| Daly et al. (2006) [30] | Ob, T2DM, HTN | UK | 3 mo | LFD | LCD | 27.5:27.5 | 102 | NA | 209.7 | 189.5 | 224.1 | NA | 46.8 | 102.0 | 36.1 | 9.1 | 142.2 | 79.5 |
| Davis et al. (2009) [31] | Ob, T2DM | US | 1 yr | LFD | LCD | 12:14.6 | 105 | 2.9 | NA | 168.3 | 124.0 | 94.8 | 48.4 | 97.1 | 36.0 | 7.5 | 127.4 | 74.9 |
| de Jonge et al. (2012) [32] | Ob | US | 2 yr | NCD | MCD | 39:39 | 811 | NA | NA | NA | NA | NA | NA | 92.9 | 32.7 | NA | NA | NA |
| de Luis et al. (2007) [33] | Ob | Spain | 3 mo | LFD | MCD | NA | 90 | NA | NA | NA | NA | NA | NA | 93.4 | 35.7 | NA | NA | NA |
| de Luis et al. (2015) [34] | Ob | Spain | 9 mo | CRD | MCD | NA | 193 | NA | 102.0 | 207.2 | 127.6 | 125.8 | 54.5 | 93.7 | 36.2 | NA | 129.0 | 81.2 |
| de Luis et al. (2015) (1) [35] | Ob | Spain | 9 mo | CRD | MCD | NA | 195 | 0 | 101.0 | 209.4 | 124.5 | 128.5 | 54.6 | 93.7 | 35.7 | NA | 129.5 | 82.1 |
| de Luis et al. (2016) [36] | Ob | Spain | 9 mo | CRD | MCD | NA | 283 | NA | 102.6 | 206.0 | 123.0 | 126.1 | 55.9 | 91.1 | 35.1 | NA | 127.3 | 81.1 |
| de Luis et al. (2019) [37] | Ob | Spain | 9 mo | CRD | LCD | NA | 270 | 0 | 103.0 | 208.5 | 123.7 | 127.7 | 55.6 | 92.0 | 34.5 | NA | 127.0 | 82.3 |
| Dyson et al. (2007) [38] | Ob, T2DM | UK | 3 mo | CRD | VLCD | 28.6:0 | 26 | NA | NA | 197.2 | 131.1 | 119.9 | 51.0 | 96.3 | 35.1 | 6.6 | NA | NA |
| Foraker et al. (2014) [39] | Ob | US | 1 yr | LFD | MCD | 0 | 79 | NA | NA | 194.2 | NA | 123.1 | 53.0 | NA | 30.3 | NA | 121.8 | 74.5 |
| Forsythe et al. (2008) [40,41] | Ob | US | 3 mo | LFD | LCD | 0 | 40 | NA | NA | 206.0 | 199.0 | 129.2 | 37.3 | NA | NA | NA | NA | NA |
| Foster et al. (2010) [42] | Ob | US | 2 yr | CRD | LCD | 32:42 | 307 | <1 | NA | 190.5 | 118.7 | 122.1 | 45.8 | 103.4 | 36.1 | NA | 124.5 | 95.0 |
| Foster et al. (2003) [43] | Ob | US | 1 yr | CRD | VLCD | 39:43 | 63 | 0 | NA | 197.3 | 127.1 | 124.9 | 48.0 | 98.5 | 34.1 | NA | 121.8 | 76.0 |
| Frisch et al. (2009) [44] | Ob | Germany | 1 yr | LFD | MCD | 20:15 | 200 | 0 | 1.0 | 213.5 | 119.6 | 137.3 | 57.2 | 99.6 | 33.7 | 5.6 | 127.0 | 86.0 |
| Gardner et al. (2007) [45] | Ob | US | 1 yr | NCD | MCD/VLCD | 2.6:22.8:11.7 | 311 | 10.0 | 94.0 | NA | 121.0 | 110.0 | 52.0 | 85.0 | 32.0 | NA | 116.0 | 75.0 |
| Goday et al. (2016) [46] | Ob, T2DM | Spain | 4 mo | CRD | VLCD | 18.2:11.1 | 89 | NA | 139.8 | 190.8 | 155.2 | 106.3 | 53.0 | 90.5 | 33.1 | 6.9 | NA | NA |
| Goldstein et al. (2011) [47] | Ob, T2DM | Israel | 1 yr | CRD | VLCD | 38.5:46.2 | 52 | NA | 188.5 | 197.0 | 200.5 | NA | 44.0 | 92.0 | 33.2 | 8.9 | 138.0 | 79.5 |
| Guldbrand et al. (2012) [48], Jonasson et al. (2014) [49] | Ob, T2DM | Sweden | 2 yr | LFD | LCD | 10:13 | 61 | NA | NA | 168.7 | 155.3 | 97.7 | 42.6 | 95.2 | 32.7 | 7.3 | 135.5 | 76.5 |
| Haufe et al. (2011,2012) [50,51] | Ob | Germany | 6 mo | LFD | LCD | 40:35 | 102 | NA | 94.5 | 87.8 | 103.6 | 117.6 | 53.4 | 92.6 | 33.0 | NA | NA | NA |
| Iqbal et al. (2010) [52] | Ob, T2DM, HTN | US | 2 yr | LFD | VLCD | 43:60 | 144 | 0.0 | 151.4 | 180.4 | 161.1 | 107.7 | 40.7 | 116.9 | 37.5 | 7.7 | 139.9 | 79.4 |
| Jabekk et al. (2010) [53] | Ob | Norway | 10 wk | SD | VLCD | 11:11 | 16 | NA | NA | NA | NA | NA | NA | 90.9 | 32.3 | NA | NA | NA |
| Jenkins et al. (2014) [54] | Ob | Canada | 7 mo | NCD | MCD | 32:50 | 39 | 4.5 | 88.2 | 263.0 | 194.9 | 174.0 | 50.3 | 84.5 | 31.1 | 5.3 | 125.1 | 76.0 |
| Keogh et al. (2007) [55] | Ob | Australia | 1 yr | CRD | LCD | NA | 25 | NA | 106.2 | 5.5 | 1.6 | 3.7 | 1.3 | 94·2 | 32·9 | NA | 122.0 | 75.0 |
| Kimura et al. (2018) [56] | T2DM | Japan | 3 mo | CRD | MCD | 4.3:4 | 48 | 100 | NA | NA | 140.3 | 114.4 | 60.5 | 62.8 | 24.8 | 7.0 | NA | NA |
| Kreider et al. (2011) [57] | Ob | US | 10 wk | SD | LCD | NA | 221 | NA | 99.8 | 199.6 | 136.3 | 120.2 | 52.8 | 90.3 | 33.8 | NA | 124.6 | 81.8 |
| Larsen et al. (2011) [58] | Ob, T2DM | Australia | 1 yr | NCD | MCD | 2.2:9.4 | 99 | NA | NA | 182.5 | 210.8 | 95.1 | 46.2 | 95.0 | NA | 7.8 | 129.8 | 81.5 |
| Liu et al. (2018) [59] | T2DM | China | 3 mo | SD | MCD | 16.3:16.3 | 122 | 100 | 150.6 | 153.9 | 116.9 | 79.9 | 56.7 | 58.5 | 21.4 | 7.1 | 105.0 | 76.6 |
| Liu et al. (2013) [60] | Ob | China | 3 mo | LFD | LCD | 4:4 | 50 | 100 | 109.4 | 197.2 | 133.8 | 130.3 | 51.8 | 65.9 | 26.7 | NA | 132.7 | 86.0 |
| Meckling et al. (2004) [61] | Ob | Canada | 10 wk | CRD | LCD | 20:25 | 40 | 0 | 105.5 | 229.0 | 135.0 | 167.0 | 50.5 | 91.7 | 32.2 | NA | 123.1 | 77.8 |
| Morris et al. (2020) [62] | Ob, T2DM | UK | 3 mo | SD | LCD | 8.3:0 | 33 | 0 | 168.8 | 166.7 | 172.7 | NA | 45.2 | 101.0 | 35.4 | 7.7 | 140.0 | 82.0 |
| Noakes et al. (2006,2010) [63,64] | Ob | Australia | 15 mo | VLFD | VLCD | 0 | 67 | NA | 95.9 | 227.4 | 145.4 | 149.3 | 49.5 | NA | 32.8 | NA | NA | NA |
| Perna et al. (2019) [65] | Ob, T2DM | Bahrain | 3 mo | CRD | LCD | 0 | 17 | 100 | 108.5 | 178.9 | 156.5 | 100.2 | 47.4 | 85.3 | 31.4 | 6.0 | NA | NA |
| Ruth et al. (2013) [66] | Ob | US | 3 mo | LFD | VLCD | 37.9:42.3 | 55 | NA | 84.0 | 182.3 | 106.5 | 109.4 | 51.6 | 99.9 | 36.5 | 5.7 | 119.9 | 76.6 |
| Saslow et al. (2017) [67] | Ob, T2DM | US | 1 yr | LFD | VLCD | 16.7:12.5 | 34 | 14.7 | NA | NA | 132.4 | 93.7 | 47.0 | 98.6 | 36.4 | 6.8 | 128.2 | 79.2 |
| Sato et al. (2017) [68] | Ob, T2DM | Japan | 6 mo | CRD | LCD | 3:9 | 66 | 100 | 126.1 | NA | 145.5 | 99.3 | 45.3 | 73.8 | 26.6 | 8.2 | NA | NA |
| Shai et al. (2008) [69], Yokose et al. (2020) [70] | Ob | Israel | 2 yr | LFD | LCD | 9.6:22.0 | 213 | NA | 91.3 | NA | 170.8 | 119.0 | 38.5 | 91.4 | 30.9 | NA | 131.3 | 79.7 |
| Soenen et al. (2012) [71] | Ob | Netherlands | 1 yr | CRD | LCD | 4.3:5.7 | 132 | NA | 97.7 | 191.4 | 143.9 | 112.1 | 52.2 | 106.7 | 36.8 | NA | 130.5 | 82.3 |
| Stern et al. (2004) [72], Samaha et al. (2003) [73] | Ob, HTN | US | 1 yr | LFD | VLCD | 36.8:31.3 | 132 | 0 | 126.1 | 186.3 | 182.2 | 115.9 | 41.0 | 130.9 | 42.9 | NA | 134.0 | 79.0 |
| Struik et al. (2020) [74], Wycherley et al. (2016) [75], Tay et al. (2015) [76], Tay et al. (2018) [77], Tay et al. (2014) [78] | Ob, T2DM, HTN | Australia | 2 yr | LFD | LCD | 18:21 | 115 | NA | 145.8 | 166.3 | 132.9 | 92.8 | 51.0 | 101.6 | 34.6 | 7.3 | 131.4 | 80.7 |
| Tsai et al. (2005) [79] | Ob | US | 1 yr | SD | VLCD | 4.4:0 | 129 | 0 | 125.9 | NA | NA | 115.0 | NA | NA | 42.9 | NA | 134.0 | NA |
| Wang et al. (2018) [80] | T2DM | China | 3 mo | LFD | MCD | 4.3:4 | 48 | 100 | NA | NA | 140.3 | 114.4 | 60.5 | 62.8 | 24.8 | 7.0 | NA | NA |
| Yancy et al. (2004) [81] | Ob | US | 6 mo | LFD | VLCD | 43.3:23.7 | 119 | NA | NA | 242.2 | 174.4 | 152.6 | 54.7 | 97.3 | 34.3 | NA | NA | NA |

FPG, fasting plasma glucose; TC, total cholesterol; TG, triglyceride; LDL-C, low-density lipoprotein cholesterol; HDL-C, high-density lipoprotein cholesterol; BW, body weight; BMI, body mass index; HbA1c, glycosylated hemoglobin; SBP, systolic blood pressure; DBP, diastolic blood pressure; Ob, obesity or overweight; LFD, low fat diet; LCD, low carbohydrate diet; NA, not available; VLCD, very-low carbohydrate diet; SD, standard diet; MCD, moderately-low carbohydrate diet; HTN, hypertension; T2DM, type 2 diabetes mellitus; NCD, normal carbohydrate diet; CRD, calorie-restricted diet; VLFD, very-low fat diet.

**Supplementary Table 4.** Characteristics of randomized controlled trials included in the meta-analysis to evaluate the effects of intermittent fasting

| Study | Population | Nationality | Duration of study | Control | Intervention | Follow-up loss rate (control:intervention), % | Subject number | Asian, % | FPG, mg/dL | TC, mg/dL | TG, mg/dL | LDL-C, mg/dL | HDL-C, mg/dL | BW, kg | BMI, kg/m^2^ | HbA1c, % | SBP, mm Hg | DBP, mm Hg |
| --- | --- | --- | --- | --- | --- | --- | --- | --- | --- | --- | --- | --- | --- | --- | --- | --- | --- | --- |
| Barnosky et al. (2017) [82,83] | Ob | US | 6 mo | CER | ADF | 17.1:26.4 | 100 | 2 | 90.0 | 187.0 | 98.0 | 111.0 | 56.0 | 96.0 | 35.0 | NA | 123.0 | 81.0 |
| Bowen et al. (2018) [84] | Ob | Australia | 4 mo | CER | ADF | 16.0:18.3 | 163 | 0 | 97.3 | 201.1 | 124.0 | 127.6 | 50.3 | 100.1 | 35.6 | NA | 120.0 | 75.2 |
| Carter et al. (2018,2016) [85,86] | Ob | Iran | 4 mo | CER | IER | 7.5:5.0 | 75 | 100 | NA | NA | NA | NA | NA | 88.3 | 31.3 | NA | 135.5 | 85.5 |
| Kunduraci et al. (2020) [87] | Ob | Turkey | 3 mo | CER | IER | 5.7:8.6 | 65 | 100 | 117 | 228.5 | 204.8 | 147.7 | 44.6 | 92.9 | 34.7 | 6.5 | 136.4 | 86.6 |
| Panizza et al. (2019) [88] | Ob | US (East Asians in Hawaii) | 3 mo | DASH | IER | 7:13 | 60 | 100 | 103.3 | 243.5 | 133.7 | 183.6 | 35.1 | 80.2 | 30.7 | NA | 133.3 | 85.2 |
| Phillips et al. (2021) [89] | Ob | Swiss | 6 mo | SD | TRE | 23.1:10.7 | 54 | 0 | 93.4 | NA | 113.3 | NA | 56.5 | 77.8 | 28.3 | 5.27 | 125 | 80.1 |
| Razavi et al. (2021) [90] | T2DM/Ob | Australia | 1 yr | CER | ADF | 31.3:27.1 | 137 | NA | 153 | 186 | 147 | 111 | 47 | 101 | 36 | 7.3 | NA | NA |
| Varady et al. (2013) [91] | Ob | US | 3 mo | SD | ADF | 6.3:6.3 | 30 | 0 | NA | 206 | 108.5 | 123 | 56.5 | 77 | 26 | NA | 121.5 | 80 |

FPG, fasting plasma glucose; TC, total cholesterol; TG, triglyceride; LDL-C, low-density lipoprotein cholesterol; HDL-C, high-density lipoprotein cholesterol; BW, body weight; BMI, body mass index; HbA1c, glycosylated hemoglobin; SBP, systolic blood pressure; DBP, diastolic blood pressure; Ob, obesity or overweight; CER, continuous energy restriction; ADF, alternate-day fasting; NA, not available; IER, intermittent energy restriction; DASH, dietary approaches to stop hypertension diet; SD, standard diet; TRE, time-restricted eating; T2DM, type 2 diabetes.

**Supplementary Table 5.** Classification of carbohydrate-restricted diets

| Diet | Carbohydrate composition |
| --- | --- |
| Normal carbohydrate diet (NCD) | >45% of 2,000 kcal/day or >255 g/day |
| Carbohydrate-restricted diet |  |
| Moderately-low or low carbohydrate diet (mLCD) |  |
| Moderately-low carbohydrate diet (MCD) | 26%–45% of 2,000 kcal/day or 130–225 g/day |
| Low carbohydrate diet (LCD) | 10%–25% of 2,000 kcal/day or 50–130 g/day |
| Very-low carbohydrate diet (VLCD) | <10% of 2,000 kcal/ day or <50 g/day |

**Supplementary Table 6.** Quality of the evidence assessment for included studies evaluating the effects of carbohydrate-restricted diets in adults with overweight/obesity: mLCD

| Quality assessment | | | | | | | No. of patients | | Effect | | Quality |
| --- | --- | --- | --- | --- | --- | --- | --- | --- | --- | --- | --- |
| No. of studies | Design | Risk of bias | Inconsistency | Indirectness | Imprecision | Other considerations | mLCD | Control | Relative (95% CI) | Absolute |  |
| BW, kg (follow-up mean 8–24 wk; better indicated by lower values) | | | | | | | | | | | |
| 24 | Randomized trials | serious | No serious inconsistency | No serious indirectness | No serious imprecise ion | Reporting bias | 1,826 | 1,834 | - | MD, 1.03 lower (1.68–0.39 lower) | ⊕⊕OO Low |
| BMI (follow-up 8–24 wk; better indicated by lower values) | | | | | | | | | | | |
| 15 | Randomized trials | Serious | No serious inconsistency | No serious indirectness | Serious | Reporting bias | 1,360 | 1,390 | - | MD, 0.23 lower (0.46 lower–0.00 higher) | ⊕OOO Very low |
| WC, cm (follow-up 12–24 wk; better indicated by lower values) | | | | | | | | | | | |
| 15 | Randomized trials | Serious | No serious inconsistency | No serious indirectness | No serious imprecision | None | 1,281 | 1,059 | - | MD, 0.65 lower (1.16–0.14 lower) | ⊕⊕⊕O Moderate |
| Fat mass, kg (better indicated by lower values) | | | | | | | | | | | |
| 14 | Randomized trials | Serious | No serious inconsistency | No serious indirectness | No serious imprecision | None | 1,059 | 1,021 | - | MD, 0.44 lower (0.83–0.04 lower) | ⊕⊕⊕O Moderate |
| Fat free mass, kg (follow-up 12–24 wk; better indicated by higher values) | | | | | | | | | | | |
| 10 | Randomized trials | Serious | No serious inconsistency | No serious indirectness | Serious | None | 585 | 554 | - | MD, 0.17 lower (0.49 lower–0.14 higher) | ⊕⊕OO Low |
| Fat mass, % (follow-up 12–24 wk; better indicated by lower values) | | | | | | | | | | | |
| 4 | Randomized trials | Serious | No serious inconsistency | No serious indirectness | Serious | None | 184 | 261 | - | MD, 0.09 higher (0.45 lower–0.64 higher) | ⊕⊕OO Low |
| SBP, mm Hg (follow-up 8–24 wk; better indicated by lower values) | | | | | | | | | | | |
| 19 | Randomized trials | Serious | No serious inconsistency | No serious indirectness | Serious | None | 1,290 | 1,322 | - | MD, 0.56 lower (1.69 lower–0.56 higher) | ⊕⊕OO Low |
| DBP, mm Hg (follow-up 8–24 wk; better indicated by lower values) | | | | | | | | | | | |
| 19 | Randomized trials | Serious | No serious inconsistency | No serious indirectness | Serious | None | 1,294 | 1,321 | - | MD, 0.69 lower (1.39 lower–0.01 higher) | ⊕⊕OO Low |
| TG, mg/dL (follow-up 8–24 wk; better indicated by lower values) | | | | | | | | | | | |
| 24 | Randomized trials | Serious | No serious inconsistency | No serious indirectness | No serious imprecision | Reporting bias | 1,437 | 1,459 | - | MD, 13.76 lower (19.78–7.74 lower) | ⊕⊕OO Low |
| LDL-C, mg/dL (follow-up 12–24 wk; better indicated by lower values) | | | | | | | | | | | |
| 21 | Randomized trials | Serious | Serious | No serious indirectness | Serious | None | 1,345 | 1,376 | - | MD, 2.29 higher (0.41 lower–4.99 higher) | ⊕OOO Very low |
| HDL-C, mg/dL (follow-up 8–24 wk; better indicated by higher values) | | | | | | | | | | | |
| 20 | Randomized trials | Serious | No serious inconsistency | No serious indirectness | No serious imprecision | None | 1,211 | 1,237 | - | MD, 2.61 higher (1.34–3.89 higher) | ⊕⊕⊕O Moderate |
| HbA1c, % (follow-up 8–24 wk; better indicated by lower values) | | | | | | | | | | | |
| 8 | Randomized trials | Serious | No serious inconsistency | No serious indirectness | Serious | None | 373 | 366 | - | MD, 0.20 lower (0.39–0.01 lower) | ⊕⊕OO Low |
| Fasting insulin, μU/mL (follow-up 12–24 wk; better indicated by lower values) | | | | | | | | | | | |
| 13 | Randomized trials | Serious | No serious inconsistency | No serious indirectness | No serious imprecision | None | 916 | 939 | - | MD, 0.94 lower (1.73–0.16 lower) | ⊕⊕⊕O Moderate |
| Fasting glucose, mg/dL (follow-up 8–24 wk; better indicated by lower values) | | | | | | | | | | | |
| 17 | Randomized trials | Serious | No serious inconsistency | No serious indirectness | Serious | None | 1,060 | 1,083 | - | MD, 0.32 lower (1.23 lower–0.58 higher) | ⊕⊕OO Low |
| CRP, mg/L (follow-up 8–24 wk; better indicated by lower values) | | | | | | | | | | | |
| 11 | Randomized trials | Serious | No serious inconsistency | No serious indirectness | No serious imprecision | Reporting bias | 701 | 690 | - | MD, 0.34 lower (0.67–0.01 lower) | ⊕⊕OO Low |
| Adiponectin, μg/mL (follow-up 8–24 wk; better indicated by lower values) | | | | | | | | | | | |
| 8 | Randomized trials | Serious | No serious inconsistency | No serious indirectness | No serious imprecision | None | 685 | 671 | - | MD, 0.45 higher (0.15–0.76 higher) | ⊕⊕⊕O Moderate |

mLCD, moderately-low or low carbohydrate diet; CI, confidence interval; BW, body weight; MD, mean difference; BMI, body mass index; WC, waist circumference; SBP, systolic blood pressure; DBP, diastolic blood pressure; TG, triglyceride; LDL-C, low-density lipoprotein cholesterol; HDL-C, high-density lipoprotein cholesterol; HbA1c, glycosylated hemoglobin; CRP, C-reactive protein.

**Supplementary Table 7.** Quality of the evidence assessment for included studies evaluating the effects of carbohydrate-restricted diets in adults with overweight/obesity: VLCD

| Quality assessment | | | | | | | No. of patients | | Effect | | Quality | Importance |
| --- | --- | --- | --- | --- | --- | --- | --- | --- | --- | --- | --- | --- |
| No of studies | Design | Risk of bias | Inconsistency | Indirectness | Imprecision | Other considerations | VLCD | Control | Relative (95% CI) | Absolute |  |  |
| BW, kg (follow-up 8–24 wk; better indicated by lower values) | | | | | | | | | | | | |
| 14 | Randomized trials | Serious | No serious inconsistency | No serious indirectness | No serious imprecision | None | 591 | 675 | - | MD, 3.67 lower (4.84–2.51 lower) | ⊕⊕⊕O Moderate |  |
| BMI, kg/m^2^ (follow-up 8–24 wk; better indicated by lower values) | | | | | | | | | | | | |
| 5 | Randomized trials | Serious | No serious inconsistency | No serious indirectness | No serious imprecision | None | 160 | 228 | - | MD, 1.88 lower (3.11–0.65 lower) | ⊕⊕⊕O Moderate |  |
| WC, cm (copy; follow-up 8–24 wk; better indicated by lower values) | | | | | | | | | | | | |
| 2 | Randomized trials | Serious | No serious inconsistency | No serious indirectness | Serious | None | 120 | 113 | - | MD, 4.11 lower (8.70 lower –0.49 higher) | ⊕⊕OO Low |  |
| Fat mass, kg (follow-up 8–24 wk; better indicated by lower values) | | | | | | | | | | | | |
| 3 | Randomized trials | Serious | No serious inconsistency | No serious indirectness | Serious | None | 85 | 83 | - | MD, 3.01 lower (6.29 lower –0.27 higher) | ⊕⊕OO Low |  |
| Fat free mass, kg (follow-up 8–24 wk; better indicated by higher values) | | | | | | | | | | | | |
| 3 | Randomized trials | Serious | No serious inconsistency | No serious indirectness | Serious | None | 85 | 83 | - | MD, 1.05 lower (1.75–0.35 lower) | ⊕⊕OO Low |  |
| Fat mass, % (follow-up 8–24 wk; better indicated by lower values) | | | | | | | | | | | | |
| 4 | Randomized trials | Serious | No serious inconsistency | No serious indirectness | No serious imprecision | None | 219 | 296 | - | MD, 1.88 lower (2.87–0.89 lower) | ⊕⊕⊕O Moderate |  |
| SBP, mm Hg (follow-up 8–24 wk; better indicated by lower values) | | | | | | | | | | | | |
| 9 | Randomized trials | Serious | No serious inconsistency | No serious indirectness | No serious imprecision | None | 4 | 502 | - | MD, 1.97 lower (3.68–0.25 lower) | ⊕⊕⊕O Moderate |  |
| DBP, mm Hg (follow-up 8–24 wk; better indicated by lower values) | | | | | | | | | | | | |
| 9 | Randomized trials | Serious | No serious inconsistency | No serious indirectness | Serious | None | 404 | 502 | - | MD, 0.68 lower (1.79 lower –0.44 higher) | ⊕⊕OO Low |  |
| TG, mg/dL (follow-up 8–24 wk; better indicated by lower values) | | | | | | | | | | | | |
| 13 | Randomized trials | Serious | No serious inconsistency | No serious indirectness | No serious imprecision | Reporting bias | 488 | 571 | - | MD, 21.33 lower (30.46–12.21 lower) | ⊕⊕OO Low |  |
| LDL-C, mg/dL (follow-up 8–24 wk; better indicated by lower values) | | | | | | | | | | | | |
| 12 | Randomized trials | Serious | No serious inconsistency | No serious indirectness | No serious imprecision | None | 469 | 554 | - | MD, 7.52 higher (3.34–11.70 higher) | ⊕⊕⊕O Moderate |  |
| HDL-C, mg/dL (follow-up 8–24 wk; better indicated by higher values) | | | | | | | | | | | | |
| 13 | Randomized trials | Serious | Serious | No serious indirectness | No serious imprecision | None | 488 | 570 | - | MD, 30 higher (1.79–6.82 higher) | ⊕⊕OO Low |  |
| HbA1c, % (follow-up 8–24 wk; better indicated by lower values) | | | | | | | | | | | | |
| 6 | Randomized trials | Serious | No serious inconsistency | No serious indirectness | Serious | None | 179 | 175 | - | MD, 0.23048 lower (2.87 lower–0.01 higher) | ⊕⊕OO Low |  |
| Fasting insulin, μU/mL (follow-up 8–24 wk; better indicated by lower values) | | | | | | | | | | | | |
| 6 | Randomized trials | Serious | No serious inconsistency | No serious indirectness | Serious | None | 255 | 348 | - | MD, 1.37 lower (2.89 lower –0.15 higher) | ⊕⊕OO Low |  |
| Fasting glucose, mg/dL (follow-up 8–24 wk; better indicated by lower values) | | | | | | | | | | | | |
| 9 | Randomized trials | Serious | No serious inconsistency | No serious indirectness | Serious | None | 382 | 348 | - | MD, 0.44 lower (2.66 lower –1.78 higher) | ⊕⊕OO Low |  |
| CRP, mg/L (follow-up 8–24 wk; better indicated by lower values) | | | | | | | | | | | | |
| 5 | Randomized trials | Serious | No serious inconsistency | No serious indirectness | Serious | None | 178 | 193 | - | MD, 0.63 lower (1.41 lower –0.15 higher) | ⊕⊕OO Low |  |
| Adiponectin, μg/mL (follow-up 8–24 wk; better indicated by higher values) | | | | | | | | | | | | |
| 2 | Randomized trials | Serious | No serious inconsistency | No serious indirectness | Serious | None | 93 | 88 | - | MD, 0.75 higher (0.29–1.21 higher) | ⊕⊕OO Low |  |

VLCD, very-low carbohydrate diet; CI, confidence interval; BW, body weight; MD, mean difference; BMI, body mass index; WC, waist circumference; SBP, systolic blood pressure; DBP, diastolic blood pressure; TG, triglyceride; LDL-C, low-density lipoprotein cholesterol; HDL-C, high-density lipoprotein cholesterol; HbA1c, glycosylated hemoglobin; CRP, C-reactive protein.

**Supplementary Table 8.** Quality of the evidence assessment for included studies evaluating the effects of IF in adults with overweight/obesity

| Quality assessment | | | | | | | No. of patients | | Effect | | Quality | Importance |
| --- | --- | --- | --- | --- | --- | --- | --- | --- | --- | --- | --- | --- |
| No. of studies | Design | Risk of bias | Inconsistency | Indirectness | Imprecision | Other considerations | IF | Control | Relative (95% CI) | Absolute |  |  |
| HbA1c (follow-up 12–24 wk; better indicated by lower values) | | | | | | | | | | | | |
| 3 | Randomized trials | Serious | No serious inconsistency | Serious | Serious | None | 88 | 85 | - | MD, 0.11 higher (0.04 lower–0.26 higher) | ⊕OOO Very low |  |
| Fasting glucose, mg/dL (follow-up 12–24 wk; better indicated by lower values) | | | | | | | | | | | | |
| 5 | Randomized trials | No serious risk of bias | No serious inconsistency | Serious | Serious | None | 179 | 180 | - | MD, 0.89 lower (4.3 lower–2.53 higher) | ⊕⊕OO Low |  |
| Fasting insulin, μU/mL (follow-up 12–24 wk; better indicated by lower values) | | | | | | | | | | | | |
| 4 | Randomized trials | No serious risk of bias | No serious inconsistency | Serious | Serious | None | 154 | 160 | - | MD, 0.43 lower (1.99 lower–1.14 higher) | ⊕⊕OO Low |  |
| BW, kg (follow-up 12–24 wk; better indicated by lower values) | | | | | | | | | | | | |
| 8 | Randomized trials | Serious | No serious inconsistency | Serious | Serious | None | 274 | 280 | - | MD, 1.22 lower (3.49 lower–1.05 higher) | ⊕OOO Very low |  |
| HOMA-IR (follow-up 12–24 wk; better indicated by lower values) | | | | | | | | | | | | |
| 2 | Randomized trials | Serious | No serious inconsistency | Serious | Serious | None | 57 | 62 | - | MD, 0.22 lower (1.48 lower–1.05 higher) | ⊕OOO Very low |  |
| BMI, kg/m^2^ (follow-up 12–24 wk; better indicated by lower values) | | | | | | | | | | | | |
| 5 | Randomized trials | No serious risk of bias | No serious inconsistency | Serious | Serious | None | 192 | 188 | - | MD, 0.49 lower (1.13 lower–0.14 higher) | ⊕OOO Very low |  |
| BW, kg (follow-up 12–24 wk; better indicated by lower values) | | | | | | | | | | | | |
| 8 | Randomized trials | No serious risk of bias | No serious inconsistency | Serious | Serious | None | 274 | 280 | - | MD, 1.22 lower (3.49 lower–1.05 higher) | ⊕⊕OO Low |  |
| WC, cm (follow-up 12–24 wk; better indicated by lower values) | | | | | | | | | | | | |
| 3 | Randomized trials | Serious | No serious inconsistency | Serious | Serious | None | 93 | 87 | - | MD, 1.95 lower (4.09 lower–0.2 higher) | ⊕OOO Very low |  |
| SBP, mm Hg (follow-up 12–24 wk; better indicated by lower values) | | | | | | | | | | | | |
| 6 | Randomized trials | No serious risk of bias | Serious | Serious | Serious | None | 203 | 201 | - | MD, 0.87 higher (2.56 lower–4.39 higher) | ⊕OOO Very low |  |
| DBP, mm Hg (follow-up 12–24 wk; better indicated by lower values) | | | | | | | | | | | | |
| 6 | Randomized trials | No serious risk of bias | Serious | Serious | Serious | None | 203 | 201 | - | MD, 0.16 lower (2.89 lower–2.56 higher) | ⊕OOO Very low |  |
| Fat free mass, kg (follow-up 12–24 wk; better indicated by lower values) | | | | | | | | | | | | |
| 8 | Randomized trials | Serious | No serious inconsistency | Serious | Serious | None | 269 | 271 | - | MD, 0.36 lower (0.87 lower–0.16 higher) | ⊕OOO Very low |  |
| Fat mass, kg (follow-up 12–24 wk; better indicated by lower values) | | | | | | | | | | | | |
| 8 | Randomized trials | Serious | No serious inconsistency | Serious | Serious | None | 269 | 271 | - | MD, 0.67 lower (1.95 lower–0.62 higher) | ⊕OOO Very low |  |
| Fat mass, % (follow-up 12–24 wk; better indicated by lower values) | | | | | | | | | | | | |
| 3 | Randomized trials | Serious | No serious inconsistency | Serious | Serious | None | 73 | 69 | - | MD, 0.27 higher (0.48 lower–1.01 higher) | ⊕OOO Very low |  |
| HDL-C, mg/dL (follow-up 12–24 wk; better indicated by higher values) | | | | | | | | | | | | |
| 6 | Randomized trials | Serious | No serious inconsistency | Serious | Serious | None | 218 | 214 | - | MD, 0.17 lower (3.27 lower–2.89 higher) | ⊕OOO Very low |  |
| LDL-C, mg/dL (follow-up 12–24 wk; better indicated by lower values) | | | | | | | | | | | | |
| 5 | Randomized trials | Serious | No serious inconsistency | Serious | Serious | None | 193 | 194 | - | MD, 0.24 lower (5.08 lower–4.59 higher) | ⊕OOO Very low |  |
| TG, mg/dL (follow-up 12–24 wk; better indicated by lower values) | | | | | | | | | | | | |
| 6 | Randomized trials | Serious | No serious inconsistency | Serious | Serious | None | 218 | 214 | - | MD, 1.51 lower (17.06 lower–14.04 higher) | ⊕OOO Very low |  |
| HbA1c (Copy; follow-up 12–24 wk; better indicated by lower values) | | | | | | | | | | | | |
| 3 | Randomized trials | Serious | No serious inconsistency | Serious | Serious | None | 88 | 85 | - | MD, 0.11 higher (0.04 lower–0.26 higher) | ⊕OOO Very low |  |
| BW, kg (Copy; follow-up 12–24 wk; better indicated by lower values) | | | | | | | | | | | | |
| 8 | Randomized trials | Serious | No serious inconsistency | Serious | Serious | None | 274 | 280 | - | MD, 1.22 lower (3.49 lower–1.05 higher) | ⊕OOO Very low |  |

IF, intermittent fasting; CI, confidence interval; HbA1c, glycosylated hemoglobin; MD, mean difference; BW, body weight; HOMA-IR, homeostatic model assessment for insulin resistance; BMI, body mass index; WC, waist circumference; SBP, systolic blood pressure; DBP, diastolic blood pressure; HDL-C, high-density lipoprotein cholesterol; LDL-C, low-density lipoprotein cholesterol; TG, triglyceride.

**Supplementary Table 9.** Quality of the evidence assessment for included studies evaluating the effects of carbohydrate-restricted diets in adults with diabetes: mLCD

| Quality assessment | | | | | | | No. of patients | | Effect | | Quality | Importance |
| --- | --- | --- | --- | --- | --- | --- | --- | --- | --- | --- | --- | --- |
| No. of studies | Design | Risk of bias | Inconsistency | Indirectness | Imprecision | Other considerations | mLCD | Control | Relative (95% CI) | Absolute |  |  |
| HbA1c (follow-up 8–24 wk; better indicated by lower values) | | | | | | | | | | | | |
| 10 | Randomized trials | Serious | No serious inconsistency | No serious indirectness | No serious imprecision | None | 383 | 375 | - | MD, 0.21 lower (0.32–0.10 lower) | ⊕⊕⊕O Moderate |  |
| HOMA-IR (follow-up 8–24 wk; better indicated by lower values) | | | | | | | | | | | | |
| 10 | Randomized trials | Serious | No serious inconsistency | Serious | Serious | Reporting bias | 128 | 120 | - | MD, 0.53 lower (0.96–0.11 lower) | ⊕OOO Very low |  |
| Fasting glucose (follow-up 8–24 wk; better indicated by lower values) | | | | | | | | | | | | |
| 6 | Randomized trials | Serious | No serious inconsistency | No serious indirectness | Serious | None | 172 | 165 | - | MD, 9.88 lower (18.04–1.71 lower) | ⊕⊕OO Low |  |
| Fasting insulin (follow-up 8–24 wk; better indicated by lower values) | | | | | | | | | | | | |
| 3 | Randomized trials | Serious | No serious inconsistency | No serious indirectness | Serious | None | 128 | 120 | - | MD, 2.11 lower (3.70–0.52 lower) | ⊕⊕OO Low |  |
| BW (follow-up 8–24 wk; better indicated by lower values) | | | | | | | | | | | | |
| 8 | Randomized trials | Serious | No serious inconsistency | No serious indirectness | Serious | None | 320 | 299 | - | MD, 1.54 lower (3.11 lower–0.02 higher) | ⊕⊕OO Low |  |
| SBP, mm Hg (follow-up 8–24 wk; better indicated by lower values) | | | | | | | | | | | | |
| 6 | Randomized trials | Serious | No serious inconsistency | No serious indirectness | No serious imprecision | None | 262 | 248 | - | MD, 2.99 lower (5.48–0.49 lower) | ⊕⊕⊕O Moderate |  |
| DBP, mm Hg (follow-up 8–24 wk; better indicated by lower values) | | | | | | | | | | | | |
| 6 | Randomized trials | Serious | No serious inconsistency | No serious indirectness | Serious5 | None | 266 | 247 | - | MD, 1.07 lower (2.43 lower–0.29 higher) | ⊕⊕OO Low |  |
| TG, mg/dL (follow-up 8–24 wk; better indicated by lower values) | | | | | | | | | | | | |
| 10 | Randomized trials | Serious | No serious inconsistency | No serious indirectness | No serious imprecision | Reporting bias | 380 | 362 | - | MD, 17.22 lower (34.27–0.18 lower) | ⊕⊕OO Low |  |
| LDL-C, mg/dL (follow-up 8–24 wk; better indicated by lower values) | | | | | | | | | | | | |
| 8 | Randomized trials | Serious | No serious inconsistency | No serious indirectness | Serious | None | 308 | 299 | - | MD, 0.35 higher (3.03 lower to 3.72 higher) | ⊕⊕OO Low |  |
| HDL-C, mg/dL (follow-up 8–24 wk; better indicated by lower values) | | | | | | | | | | | | |
| 8 | Randomized trials | Serious | No serious inconsistency | No serious indirectness | No serious imprecision | None | 283 | 264 | - | MD, 2.30 higher (0.23–4.37 higher) | ⊕⊕⊕O Moderate |  |

mLCD, moderately-low or low carbohydrate diet; CI, confidence interval; HbA1c, glycosylated hemoglobin; MD, mean difference; HOMA-IR, homeostatic model assessment for insulin resistance; BW, body weight; SBP, systolic blood pressure; DBP, diastolic blood pressure; TG, triglyceride; LDL-C, low-density lipoprotein cholesterol; HDL-C, high-density lipoprotein cholesterol.

**Supplementary Table 10.** Quality of the evidence assessment for included studies evaluating the effects of carbohydrate-restricted diets in adults with diabetes: VLCD

| Quality assessment | | | | | | | No. of patients | | Effect | | Quality | Importance |
| --- | --- | --- | --- | --- | --- | --- | --- | --- | --- | --- | --- | --- |
| No. of studies | Design | Risk of bias | Inconsistency | Indirectness | Imprecision | Other considerations | VLCD | Control | Relative (95% CI) | Absolute |  |  |
| HbA1c (follow-up mean 24 wk; better indicated by lower values) | | | | | | | | | | | | |
| 5 | Randomized trials | Serious | No serious inconsistency | No serious indirectness | No serious imprecision | None | 161 | 160 | - | MD, 0.32 lower (0.57–0.06 lower) | ⊕⊕⊕O Moderate |  |
| HOMA-IR (follow-up mean 12–24 wk; better indicated by lower values) | | | | | | | | | | | | |
| 2 | Randomized trials | Serious | No serious inconsistency | No serious indirectness | Serious | None | 61 | 58 | - | MD, 1.07 lower (3.13 lower–0.98 higher) | ⊕⊕OO Low |  |
| Fasting glucose (follow-up mean 12–24 wk; better indicated by lower values) | | | | | | | | | | | | |
| 3 | Randomized trials | Serious | No serious inconsistency | No serious indirectness | Serious | None | 135 | 132 | - | MD, 9.64 lower (19.54 lower–0.26 higher) | ⊕⊕OO Low |  |
| BW (follow-up mean 12–24 wk; better indicated by lower values) | | | | | | | | | | | | |
| 4 | Randomized trials | Serious | No serious inconsistency | No serious indirectness | No serious imprecision | None | 147 | 144 | - | MD, 3.84 lower (7.55–0.13 lower) | ⊕⊕⊕O Moderate |  |
| Fasting insulin (better indicated by lower values) | | | | | | | | | | | | |
| 1 | Randomized trials | Serious | No serious inconsistency | No serious indirectness | Serious | None | 16 | 18 | - | MD, 2.80 lower (5.84 lower–0.24 higher) | ⊕⊕OO Low |  |
| SBP, mm Hg (better indicated by lower values) | | | | | | | | | | | | |
| 3 | Randomized trials | Serious | No serious inconsistency | No serious indirectness | Serious | None | 106 | 112 | - | MD, 0.34 higher (3.61 lower–4.28 higher) | ⊕⊕OO Low |  |
| DBP, mm Hg (better indicated by lower values) | | | | | | | | | | | | |
| 3 | Randomized trials | Serious | No serious inconsistency | No serious indirectness | Serious | None | 106 | 112 | - | MD, 1.38 higher (0.90 lower–3.67 higher) | ⊕⊕OO Low |  |
| TG, mg/dL (better indicated by lower values) | | | | | | | | | | | | |
| 5 | Randomized trials | Serious | No serious inconsistency | No serious indirectness | Serious | None | 158 | 155 | - | MD, 11.40 lower (27.01 lower–4.22 higher) | ⊕⊕OO Low |  |
| LDL-C, mg/dL (better indicated by lower values) | | | | | | | | | | | | |
| 4 | Randomized trials | Serious | No serious inconsistency | No serious indirectness | No serious imprecision | None | 139 | 138 | - | MD, 7.19 higher (0.02–14.36 higher) | ⊕⊕⊕O Moderate |  |
| HDL-C, mg/dL (better indicated by lower values) | | | | | | | | | | | | |
| 5 | Randomized trials | Serious | No serious inconsistency | No serious indirectness | Serious | None | 158 | 154 | - | MD, 0.43 higher (1.98 lower–2.84 higher) | ⊕⊕OO Low |  |

VLCD, very-low carbohydrate diet; CI, confidence interval; HbA1c, glycosylated hemoglobin; MD, mean difference; HOMA-IR, homeostatic model assessment for insulin resistance; BW, body weight; SBP, systolic blood pressure; DBP, diastolic blood pressure; TG, triglyceride; LDL-C, low-density lipoprotein cholesterol; HDL-C, high-density lipoprotein cholesterol.

**Supplementary Table 11.** Quality of the evidence assessment for included studies evaluating the effects of carbohydrate-restricted diets in adults with hypertension: mLCD

| Quality assessment | | | | | | | No. of patients | | Effect | | Quality | Importance |
| --- | --- | --- | --- | --- | --- | --- | --- | --- | --- | --- | --- | --- |
| No. of studies | Design | Risk of bias | Inconsistency | Indirectness | Imprecision | Other considerations | mLCD | Control | Relative (95% CI) | Absolute |  |  |
| SBP, mm Hg (follow-up 8–24 wk; better indicated by lower values) | | | | | | | | | | | | |
| 2 | Randomized trials | Serious | No serious inconsistency | Serious | Serious | None | 97 | 98 | - | MD, 3.25 lower (7.28 lower–0.77 higher) | ⊕OOO Very low |  |
| DBP, mm Hg (follow-up 8–24 wk; better indicated by lower values) | | | | | | | | | | | | |
| 1 | Randomized trials | Serious | No serious inconsistency | Serious | Serious | None | 46 | 47 | - | MD, 1.80 lower (4.56 lower–0.96 higher) | ⊕OOO Very low |  |
| LDL-C (follow-up 8–24 wk; better indicated by lower values) | | | | | | | | | | | | |
| 1 | Randomized trials | Serious | No serious inconsistency | Serious | Serious | None | 46 | 47 | - | MD, 0.00 higher (9.55 lower–9.55 higher) | ⊕OOO Very low |  |
| TG (follow-up 8–24 wk; better indicated by lower values) | | | | | | | | | | | | |
| 2 | Randomized trials | Serious | No serious inconsistency | Serious | Serious | None | 97 | 98 | - | MD, 35.58 lower (52.84–18.33 lower) | ⊕OOO Very low |  |
| BW, kg (follow-up 8–24 wk; better indicated by lower values) | | | | | | | | | | | | |
| 2 | Randomized trials | Serious | No serious inconsistency | Serious | Serious | None | 97 | 98 | - | MD, 1.81 lower (3.93 lower–0.30 higher) | ⊕OOO Very low |  |
| HDL-C (follow-up 36–52 wk; better indicated by higher values) | | | | | | | | | | | | |
| 1 | Randomized trials | Serious | No serious inconsistency | Serious | Serious | None | 46 | 47 | - | MD, 1.60 higher (1.13 lower–4.33 higher) | ⊕OOO Very low |  |
| FMD (follow-up 36–52 wk; better indicated by lower values) | | | | | | | | | | | | |
| 1 | Randomized trials | Serious | No serious inconsistency | Serious | Serious | None | 46 | 47 | - | MD, 0.30 higher (0.58 lower–1.18 higher) | ⊕OOO Very low |  |

mLCD, moderately-low or low carbohydrate diet; CI, confidence interval; SBP, systolic blood pressure; MD, mean difference; DBP, diastolic blood pressure; LDL-C, low-density lipoprotein cholesterol; TG, triglyceride; BW, body weight; HDL-C, high-density lipoprotein cholesterol; FMD, flow-mediated dilation.

**Supplementary Table 12**. Quality of the evidence assessment for included studies evaluating the effects of carbohydrate-restricted diets in adults with hypertension: VLCD

| Quality assessment | | | | | | | No. of patients | | Effect | | Quality | Importance |
| --- | --- | --- | --- | --- | --- | --- | --- | --- | --- | --- | --- | --- |
| No. of studies | Design | Risk of bias | Inconsistency | Indirectness | Imprecision | Other considerations | VLCD | Control | Relative (95% CI) | Absolute |  |  |
| SBP, mm Hg (follow-up 8–24 wk; better indicated by lower values) | | | | | | | | | | | | |
| 2 | Randomized trials | Serious | No serious inconsistency | Serious | Serious | None | 115 | 117 | - | MD, 1.34 lower (5.20 lower–2.51 higher) | ⊕OOO Very low |  |
| DBP, mm Hg (follow-up 8–24 wk; better indicated by lower values) | | | | | | | | | | | | |
| 2 | Randomized trials | Serious | No serious inconsistency | Serious | Serious | None | 115 | 117 | - | MD, 2.01 higher (0.61 lower–4.63 higher) | ⊕OOO Very low |  |
| LDL-C (follow-up 8–24 wk; better indicated by lower values) | | | | | | | | | | | | |
| 2 | Randomized trials | Serious | Serious | Serious | Serious | None | 115 | 117 | - | MD, 8.91 higher (9.27 lower–27.08 higher) | ⊕OOO Very low |  |
| TG (follow-up 8–24 wk; better indicated by lower values) | | | | | | | | | | | | |
| 2 | Randomized trials | Serious | Serious | Serious | Serious | None | 115 | 117 | - | MD, 10.17 lower (43.00 lower–22.67 higher) | ⊕OOO Very low |  |
| BW, kg (follow-up 8–24 wk; better indicated by lower values) | | | | | | | | | | | | |
| 2 | Randomized trials | Serious | No serious inconsistency | Serious | Serious | None | 115 | 117 | - | MD, 1.16 lower (2.65 lower–0.34 higher) | ⊕OOO Very low |  |
| HDL-C (follow-up 8–24 wk; better indicated by higher values) | | | | | | | | | | | | |
| 2 | Randomized trials | Serious | Serious | Serious | Serious | None | 115 | 117 | - | MD, 1.85 higher (5.98 lower–9.69 higher) | ⊕OOO Very low |  |
| FMD (follow-up 36–52 wk; better indicated by lower values) | | | | | | | | | | | | |
| 1 | Randomized trials | Serious | No serious inconsistency | Serious | Serious | None | 26 | 23 | - | MD, 180 lower (3.48–0.12 lower) | ⊕OOO Very low |  |

VLCD, very-low carbohydrate diet; CI, confidence interval; SBP, systolic blood pressure; MD, mean difference; DBP, diastolic blood pressure; LDL-C, low-density lipoprotein cholesterol; TG, triglyceride; BW, body weight; HDL-C, high-density lipoprotein cholesterol; FMD, flow-mediated dilation.

**Supplementary Fig. 1.** Preferred reporting items for systematic reviews and meta-analyses **(**PRISMA) study flow for literature selection and exclusion process. PICO, population, intervention, comparator, outcome.

Records identified through database searching

4,979 Medline, 5,521 Embase, 7,272 Cochrane, 379 KoreaMed
18,151 Total

12,820 Records after duplicates removed

12,820 Records screened

12,166 Records excluded

654 Full-text articles assessed for eligibility

577 Full-text articles excluded, with reasons

**PICO2: intermittent fasting**

10 Studies included in quantitative synthesis (meta-analysis)

**PICO1: carbohydrate-restricted diets**

66 Studies included in quantitative synthesis (meta-analysis)

Number of studies by reason for exclusion

• 80 Inadequate population

• 99 Inadequate intervention

• 46 No intended outcomes

• 122 Inappropriate research design

• 206 Inappropriate study period (8 wk or less)

• 52 Abstracts or posters

• 5 Not English or Korean

• 2 Duplicated articles

**Supplementary Fig. 2.** Risk of bias assessment in studies evaluating the effects of carbohydrate-restricted diets in adults with overweight/obesity.


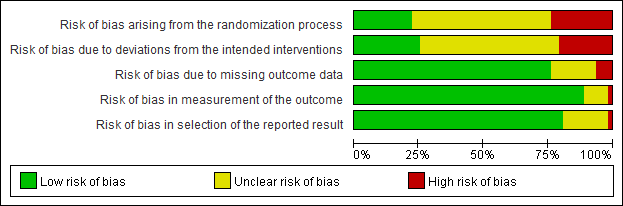

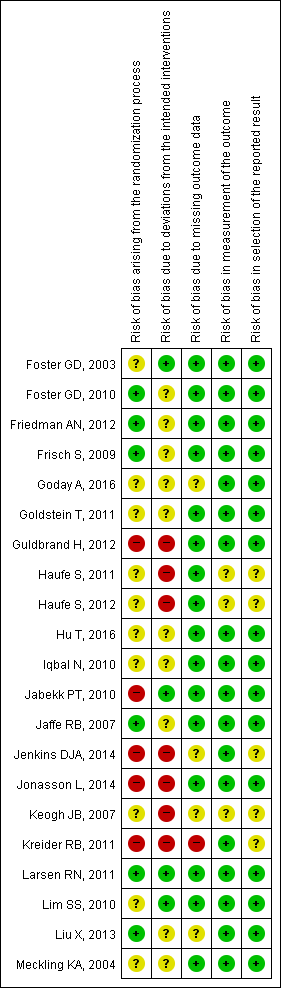

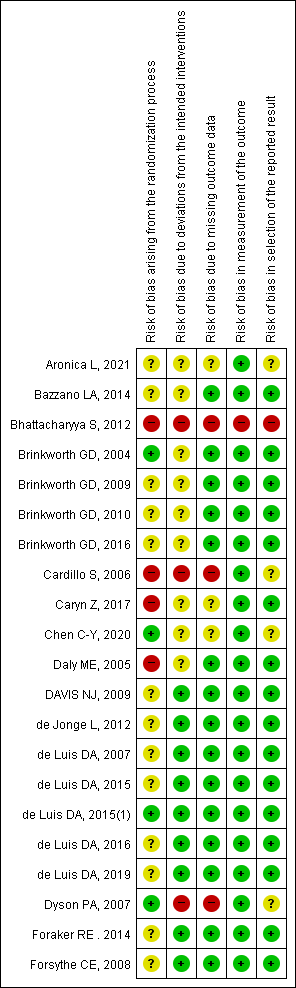

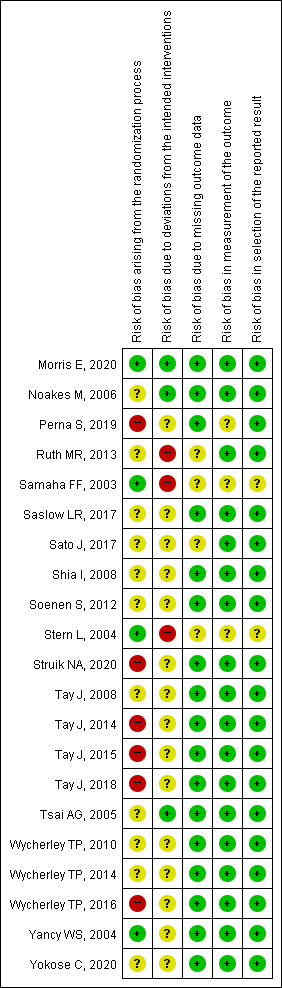


**Supplementary Fig. 3.** Effects of carbohydrate-restricted diet on body mass index (BMI) in adults with overweight/obesity. (A) Moderately-low or low carbohydrate diet (mLCD). (B) Very-low carbohydrate diet (VLCD).


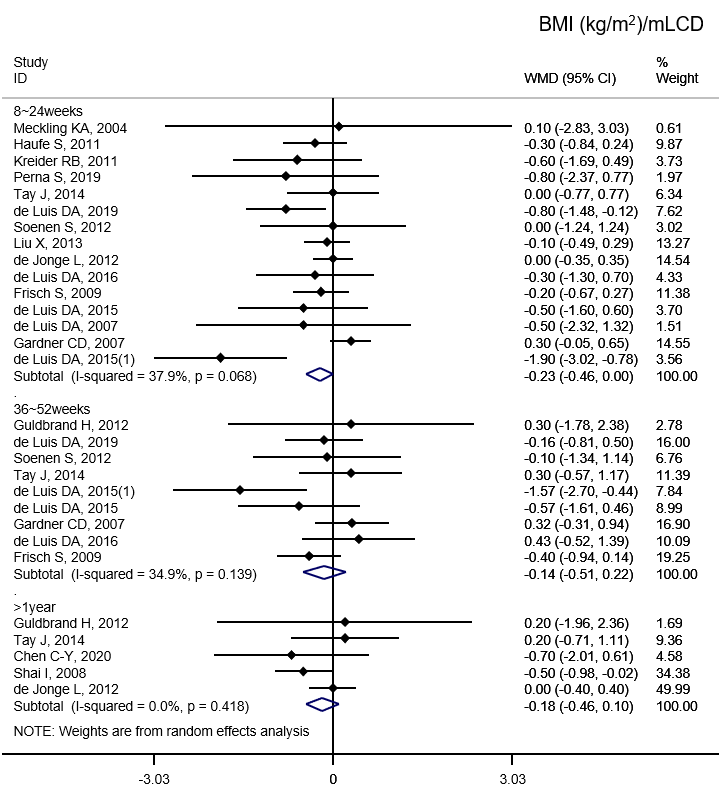

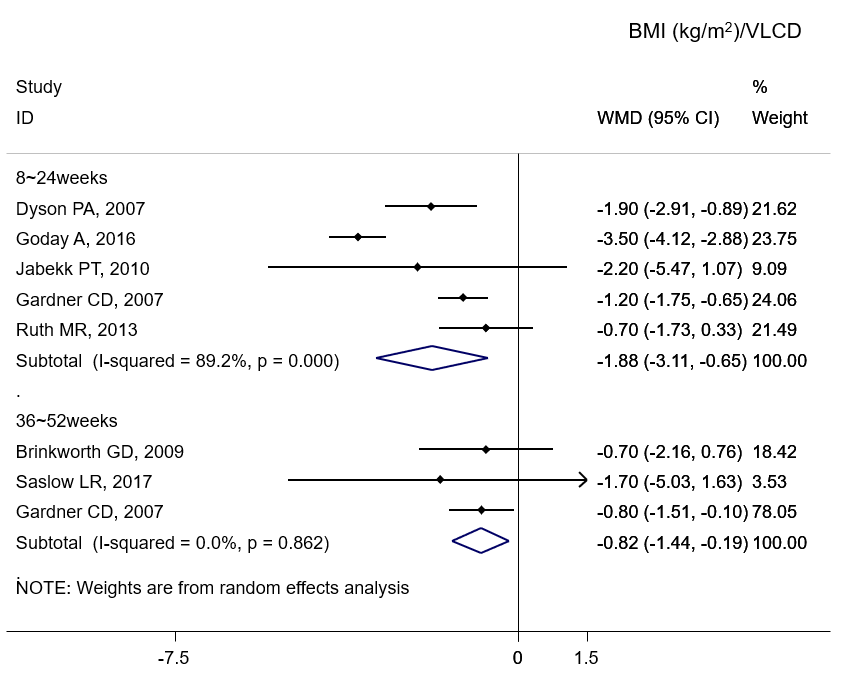


**(B)**

**(A)**

**Supplementary Fig. 4.** Effects of carbohydrate-restricted diets on waist circumference (WC) in adults with overweight/obesity. (A) Moderately-low or low carbohydrate diet (mLCD). (B) Very-low carbohydrate diet (VLCD). WMD, weighted mean difference; CI, confidence interval.

**(A)**

**(B)**

**Supplementary Fig. 5.** Effects of carbohydrate-restricted diets on fat mass in adults with overweight/obesity. (A) Moderately-low or low carbohydrate diet (mLCD). (B) Very-low carbohydrate diet (VLCD). WMD, weighted mean difference; CI, confidence interval.

**(A)**

**(B)**

**Supplementary Fig. 6.** Effects of carbohydrate-restricted diets on body fat percentage in adults with overweight/obesity. (A) Moderately-low or low carbohydrate diet (mLCD). (B) Very-low carbohydrate diet (VLCD). WMD, weighted mean difference; CI, confidence interval.


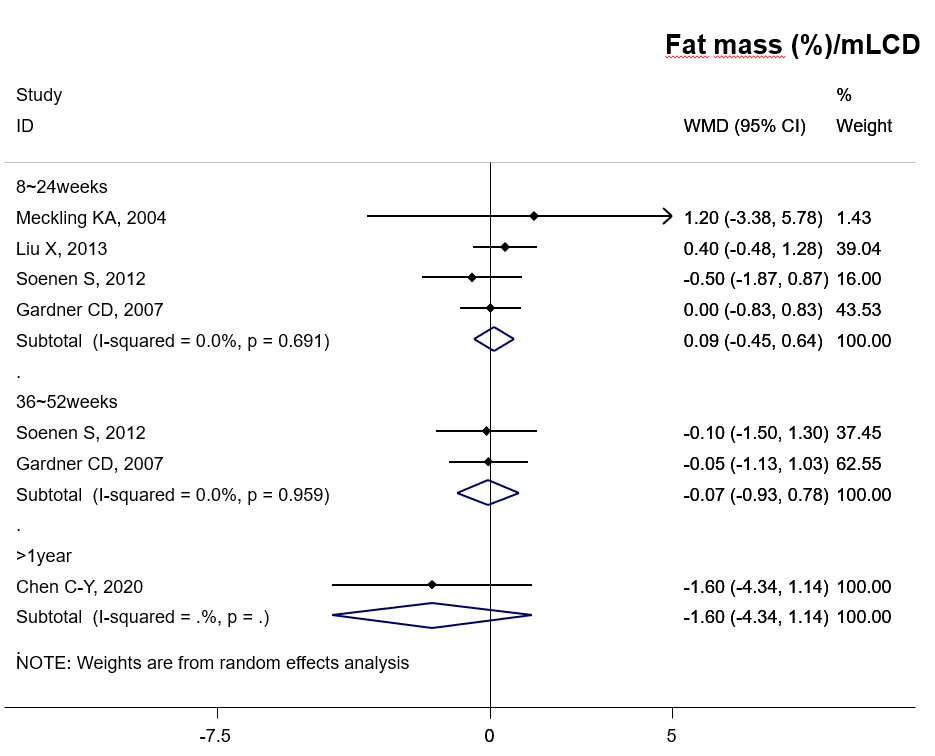


**(B)**

**(A)**


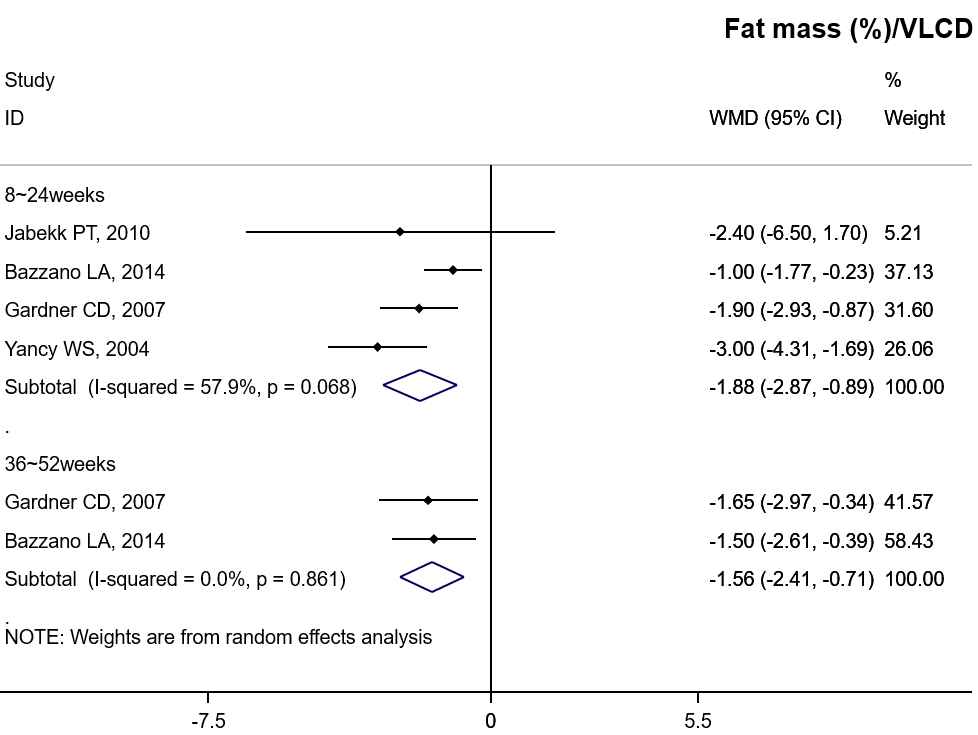


**Supplementary Fig. 7.** Effects of carbohydrate-restricted diets on serum lipid profile in adults with overweight/obesity. (A) Moderately-low or low carbohydrate diet (mLCD) on triglycerides (TG). (B) Very-low carbohydrate diet (VLCD) on TG. (C) mLCD on high-density lipoprotein cholesterol (HDL-C). (D) VLCD on HDL-C. (E) mLCD on low-density lipoprotein cholesterol (LDL-C). (F) VLCD on LDL-C. WMD, weighted mean difference; CI, confidence interval.


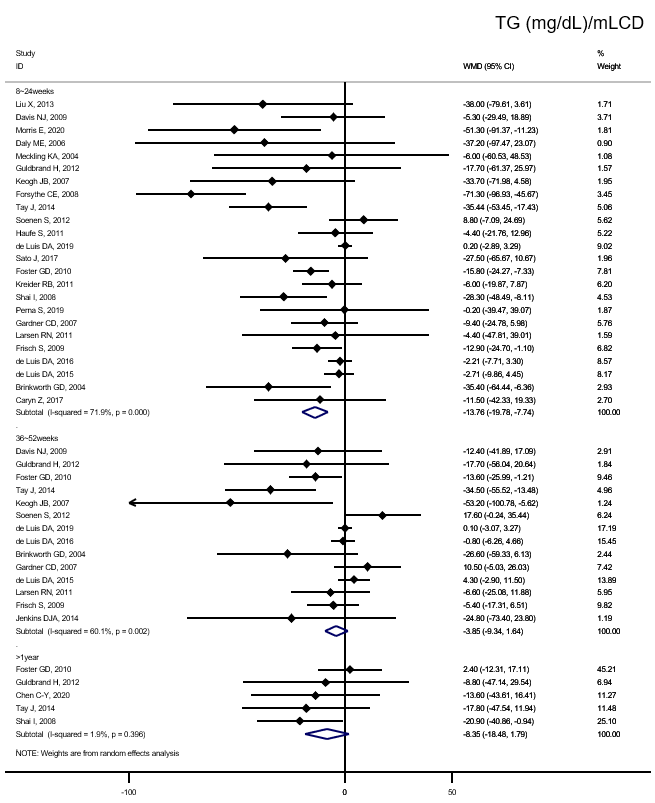


**(A)**


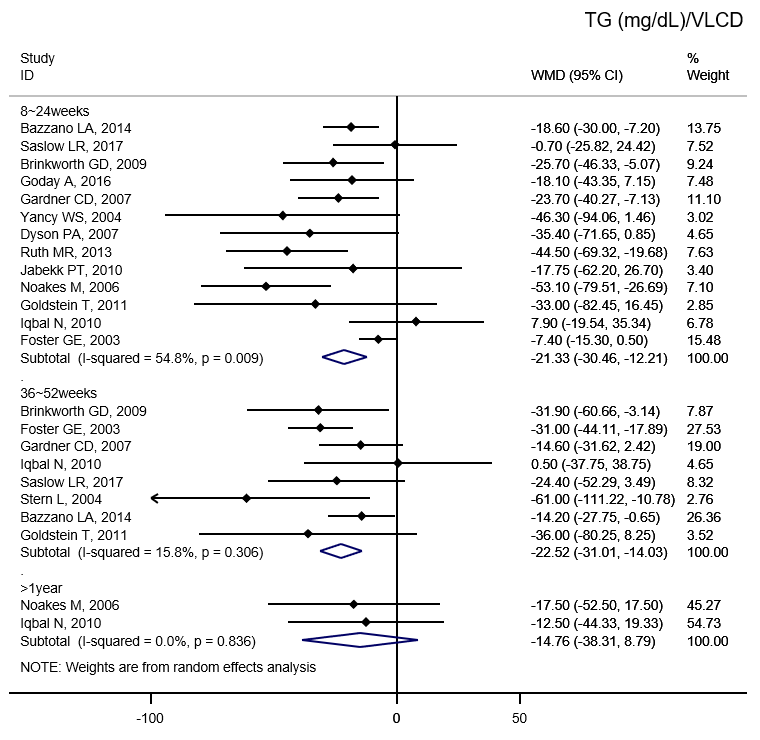


**(B)**


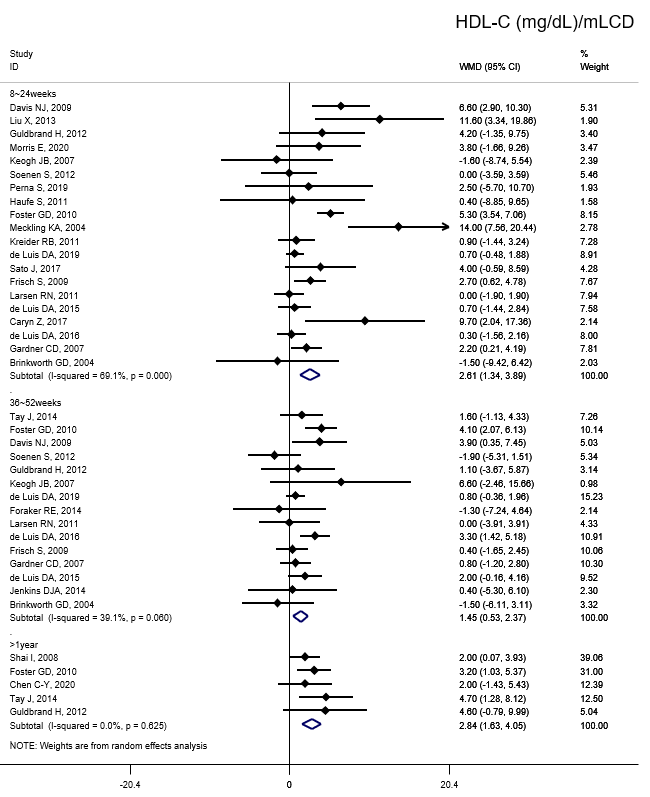


**(C)**


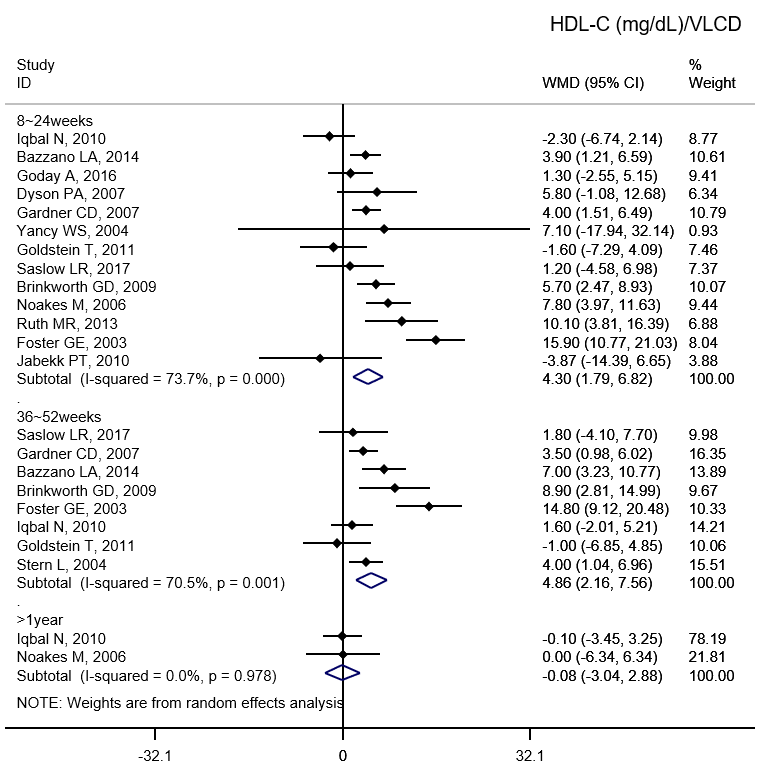


**(D)**


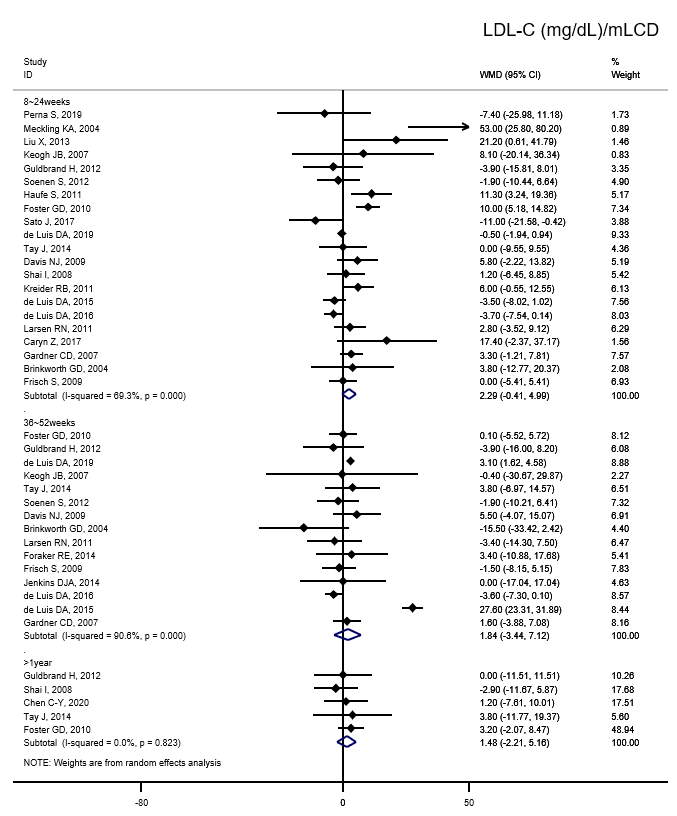


**(E)**


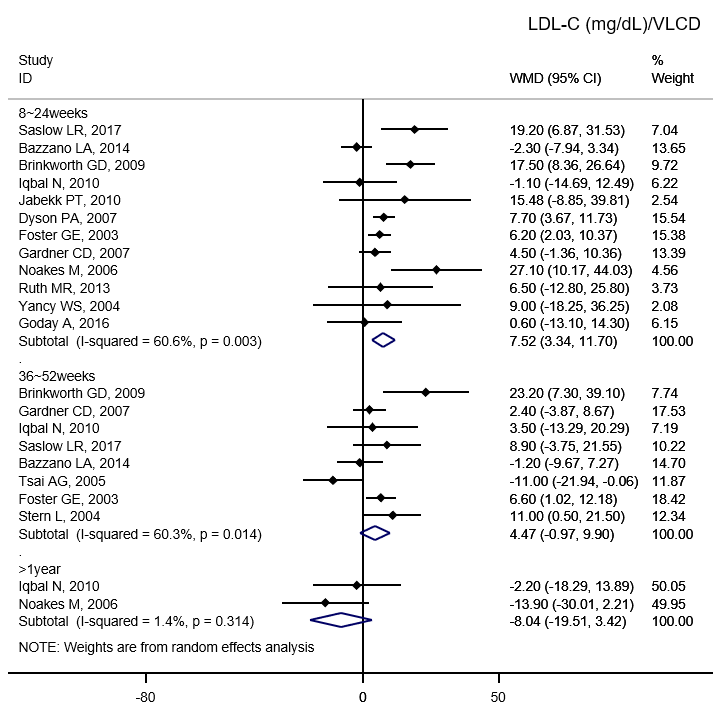


**(F)**

**Supplementary Fig. 8.** Effects of carbohydrate-restricted diets on blood pressure in adults with overweight/obesity. (A) Moderately-low or low carbohydrate diet (mLCD) on systolic blood pressure (SBP). (B) mLCD on diastolic blood pressure (DBP). (C) Very-low carbohydrate diet (VLCD) on SBP. (D) VLCD on DBP. WMD, weighted mean difference; CI, confidence interval.

**(A)**


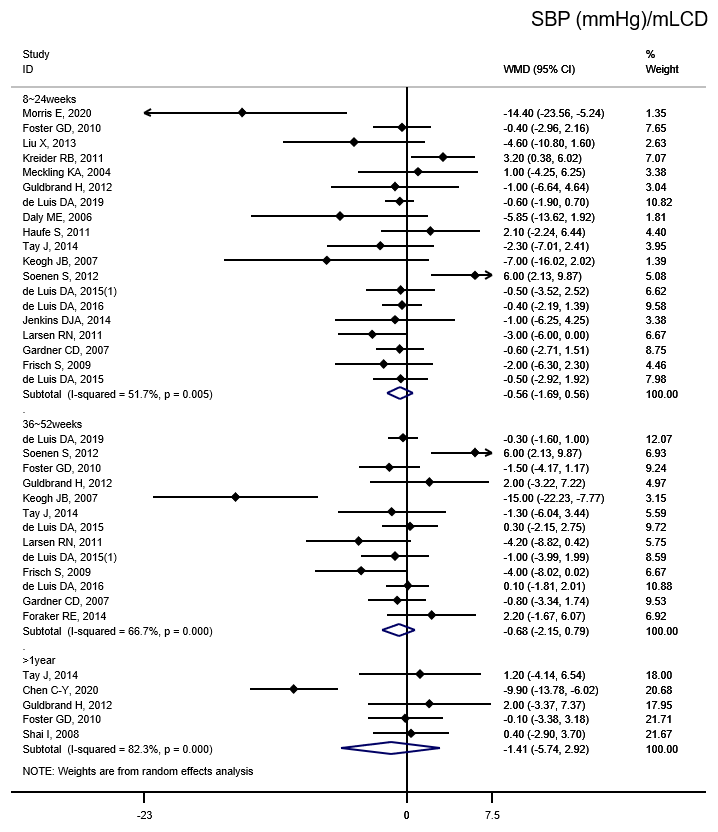


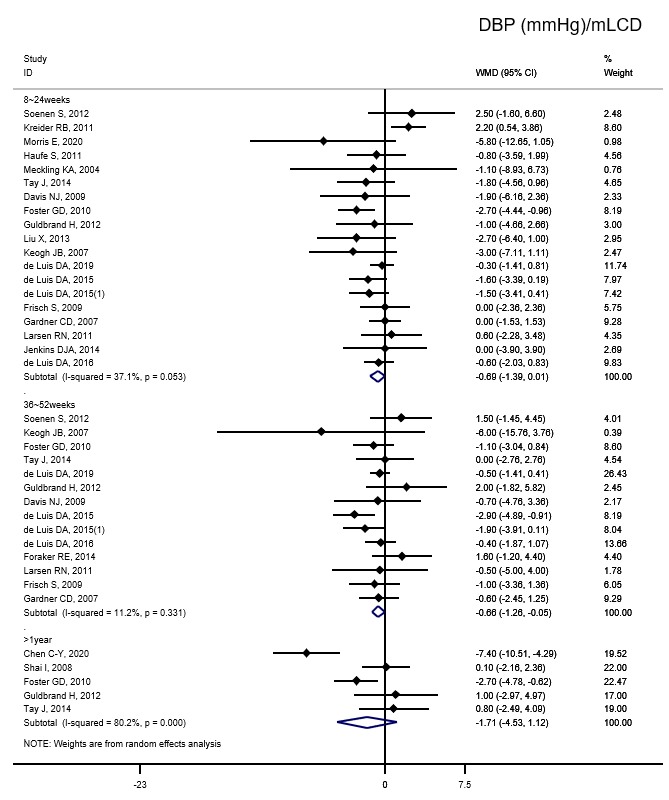


**(B)**


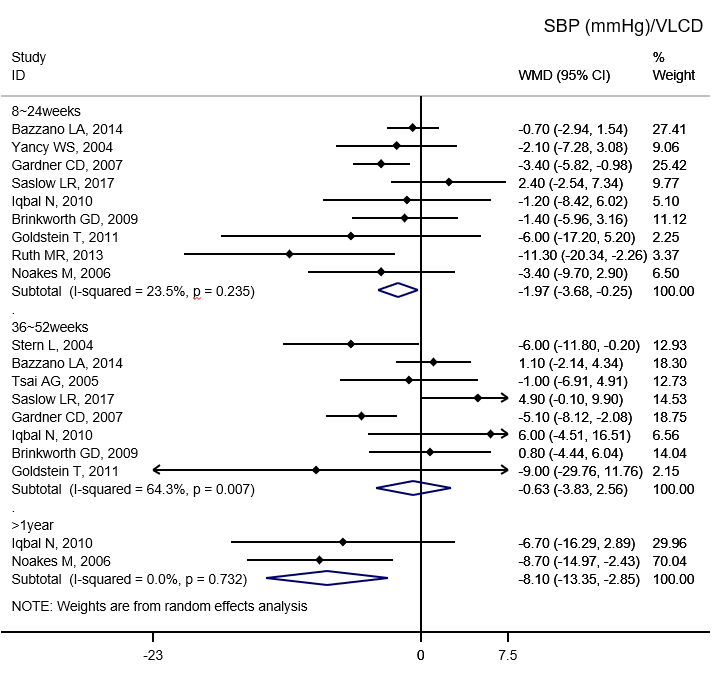


**(D)**

**(C)**


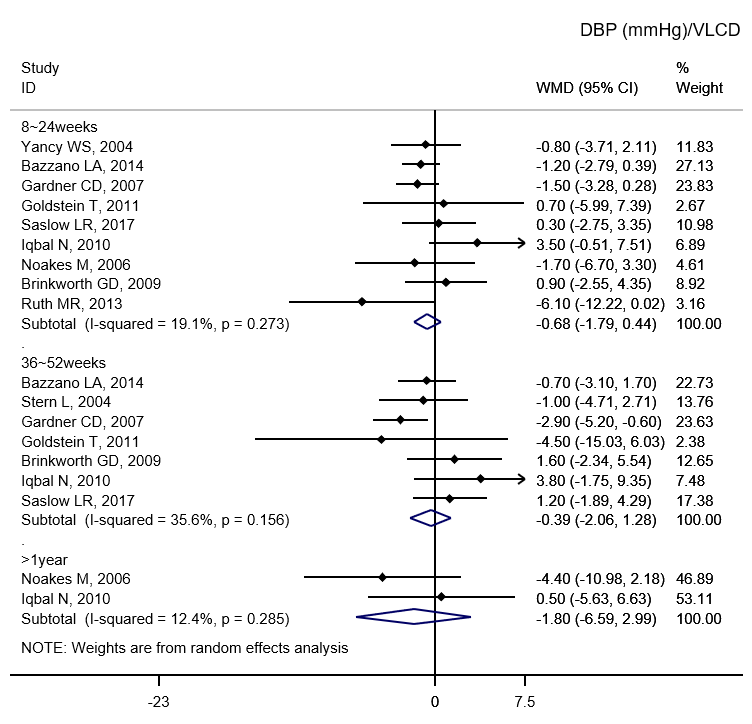


**Supplementary Fig. 9.** Effects of carbohydrate-restricted diets on fasting glucose, glycosylated hemoglobin (HbA1c), and fasting insulin levels in adults with overweight/obesity. (A) Moderately-low or low carbohydrate diet (mLCD) on fasting blood glucose. (B) Very-low carbohydrate diet (VLCD) on fasting blood glucose. (C) mLCD on HbA1c. (D) VLCD on HbA1c level. (E) mLCD on fasting insulin. (F) VLCD on fasting insulin. WMD, weighted mean difference; CI, confidence interval.


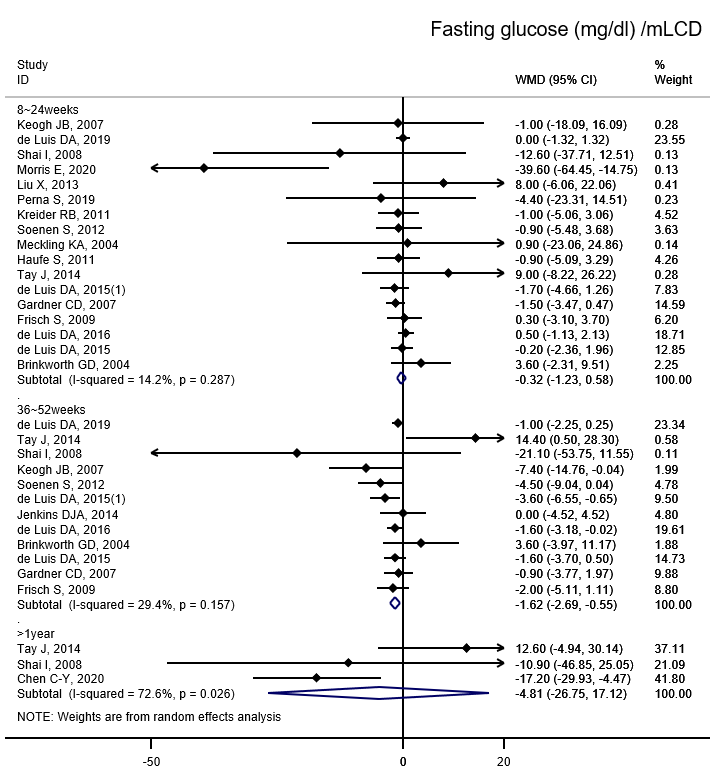


**(B)**

**(A)**


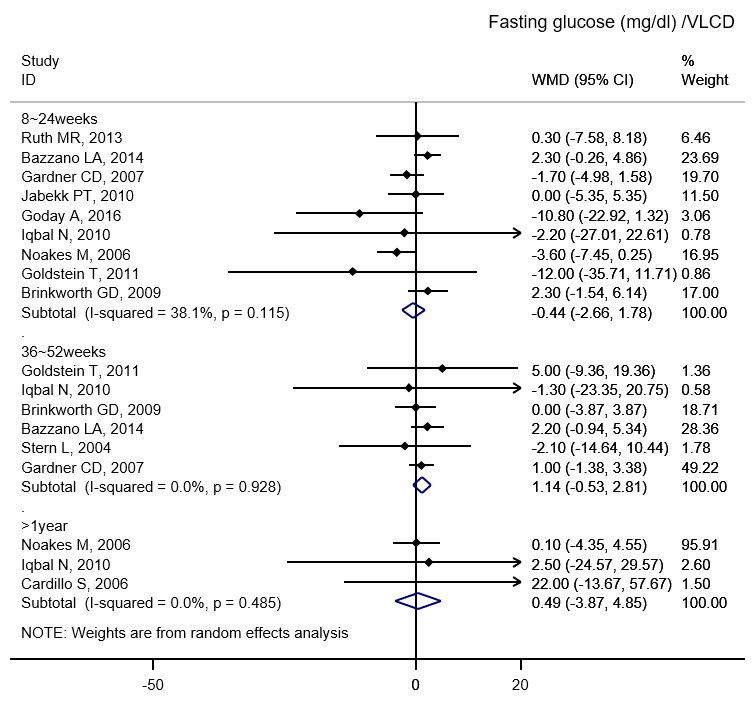

**(C)**


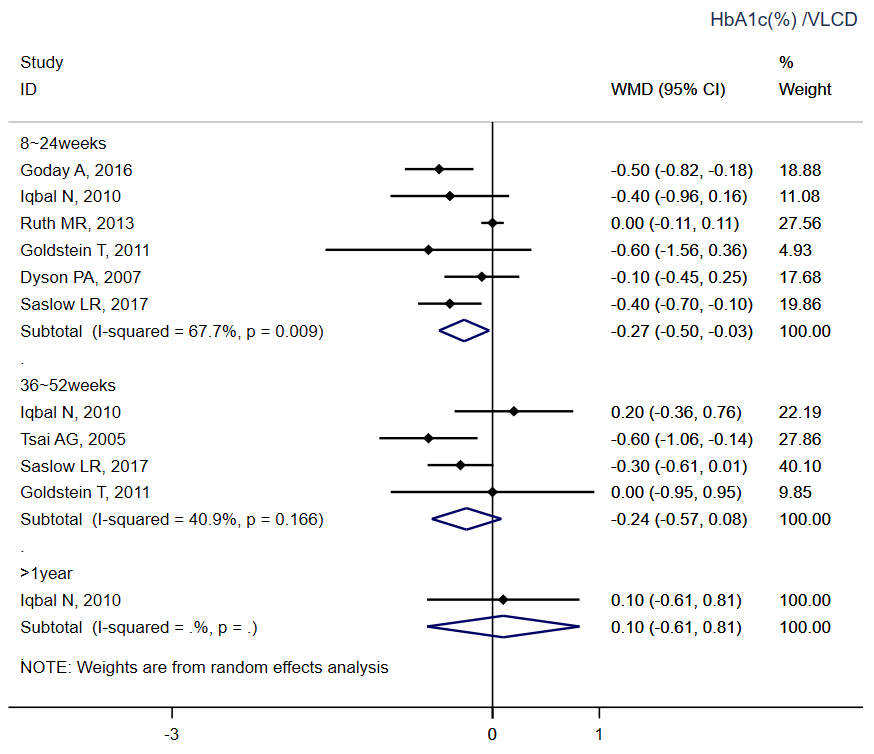


**(D)**


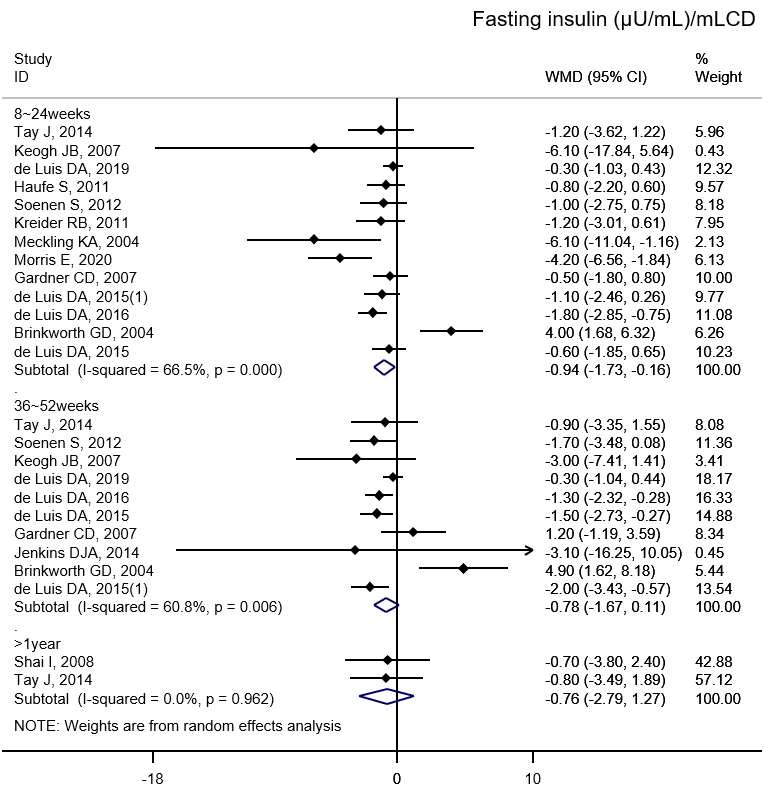


**(E)**


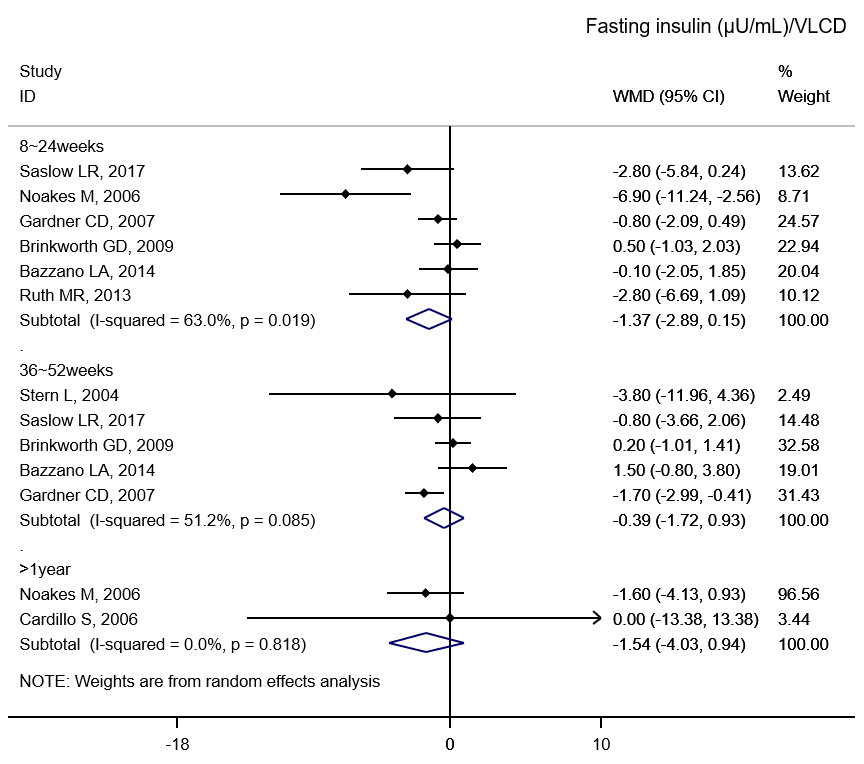


**(F)**

**Supplementary Fig. 10.** Effects of carbohydrate-restricted diets on serum adiponectin and C reactive protein (CRP) levels in adults with overweight/obesity. (A) Moderately-low or low carbohydrate diet (mLCD) on adiponectin. (B) Very-low carbohydrate diet (VLCD) on adiponectin. (C) mLCD on C-reactive protein (CRP). (D) VLCD on CRP.

**(B)**

**(A)**

**(C)**

**(D)**

**Supplementary Fig. 11.** Effects of carbohydrate-restricted diets on fat free mass in adults with overweight/obesity. (A) Moderately-low or low carbohydrate diet (mLCD). (B) Very-low carbohydrate diet (VLCD). WMD, weighted mean difference; CI, confidence interval.

**(A)**

**(B)**

**Supplementary Fig. 12.** Adverse events reported regarding carbohydrate-restricted diets in adults with overweight/obesity. (A) Nausea. (B) Vomiting. (C) Headache. (D) Constipation. CI, confidence interval.


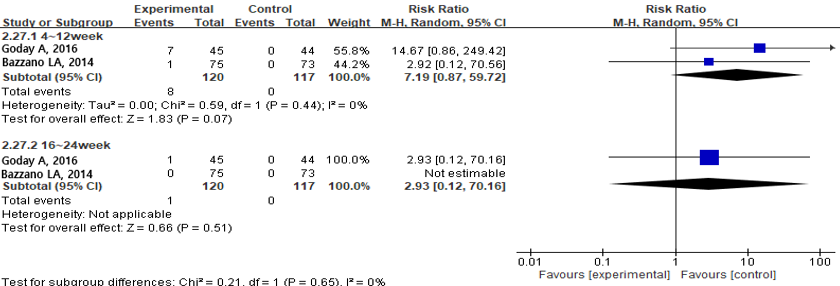

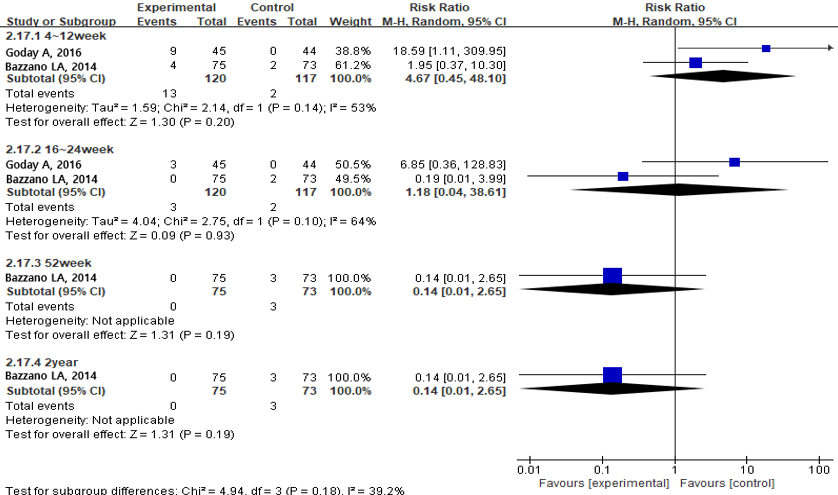


**(B)**

**(A)**


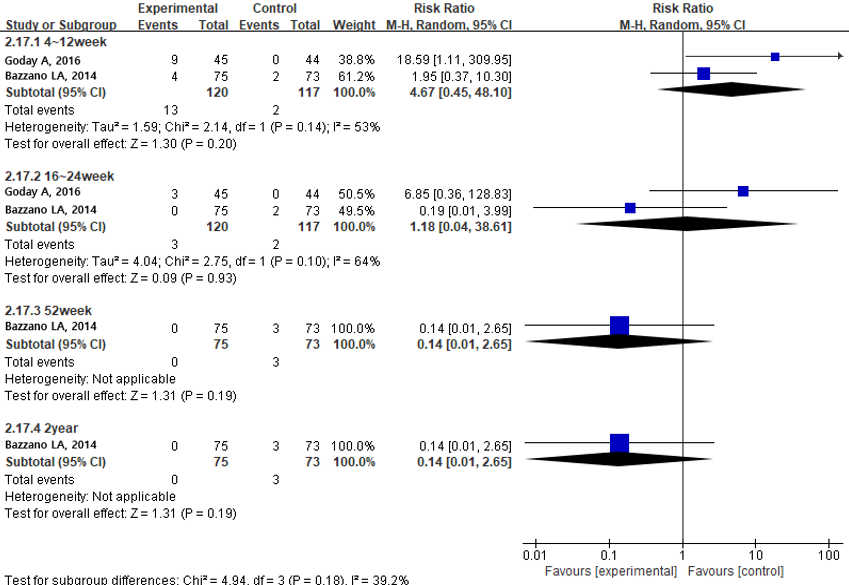

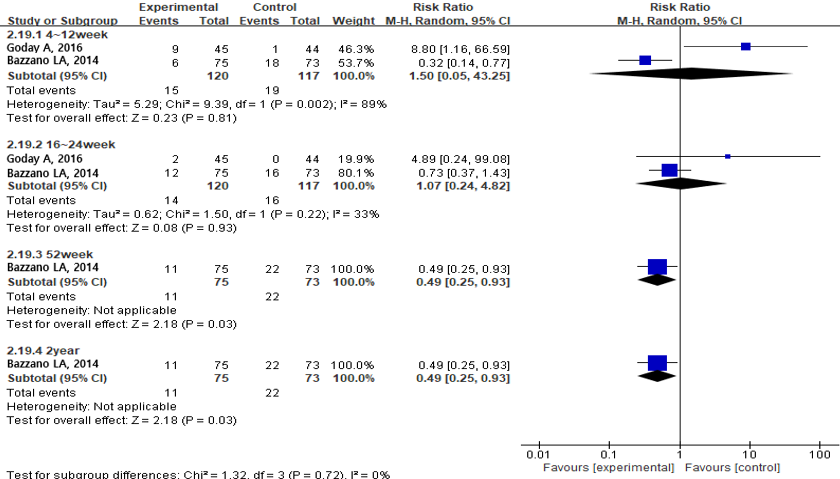


**(C)**

**(D)**

**Supplementary Fig. 13.** Risk of bias assessment in studies evaluating the effects of intermittent fasting in adults with overweight/obesity.


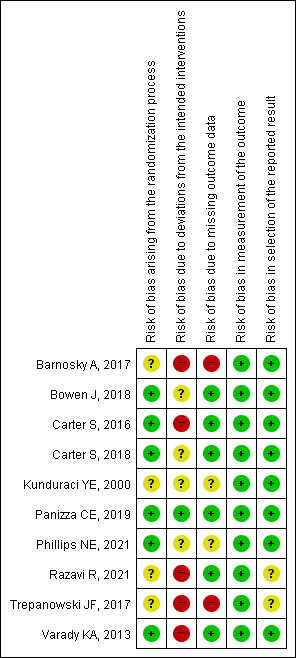

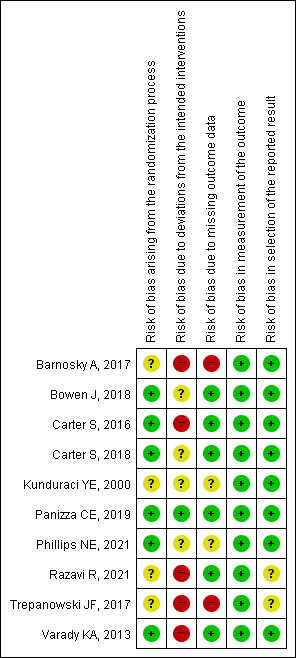


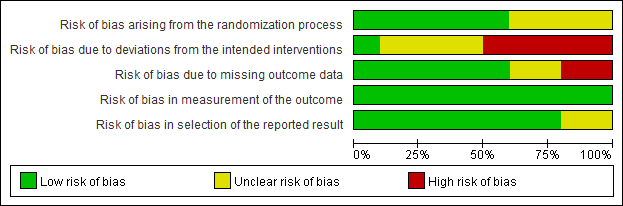


**Supplementary Fig. 14.** Effect of Intermittent fasting on (A) body weight (BW), (B) body mass index (BMI), (C) waist circumference (WC), (D) fat free mass, (E) fat mass, and (F) fat mass in adults with overweight/obesity. WMD, weighted mean difference; CI, confidence interval.

**(A)**

**(C)**

BMI (kg/m^2^)

**(B)**

**(D)**

**(E)**

**(F)**

**Supplementary Fig. 15.** Effect of Intermittent fasting on (A) triglyceride (TG; mg/dL), (B) high-density lipoprotein cholesterol (HDL-C; mg/dL), (C) low-density lipoprotein cholesterol (LDL-C; mg/dL), (D) glycosylated hemoglobin (HbA1c; %), (E) fasting glucose (mg/dL), (F) fasting insulin (μU/mL), (G) homeostatic model assessment for insulin resistance (HOMA-IR; %), (H) systolic blood pressure (SBP; mm Hg), and (I) diastolic blood pressure (DBP; mm Hg) in adults with overweight/obesity. ES, effect size; CI, confidence interval.

**(A)**

**(B)**

**(C)**

**(D)**

**(E)**

**(F)**

**(H)**

**(G)**

**SBP (mmHg)**

**DBP (mmHg)**

**(I)**

**Supplementary Fig. 16.** Risk of bias assessment in studies evaluating the effects of carbohydrate-restricted diets in adults with diabetes.


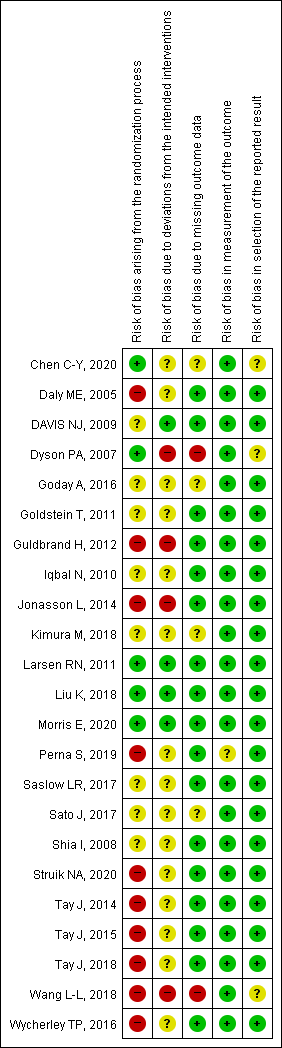

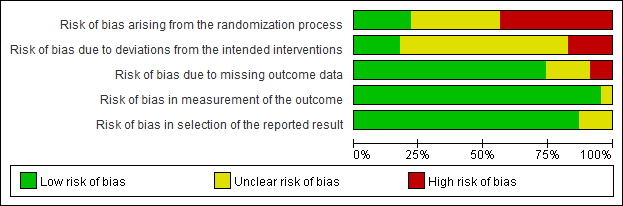


**Supplementary Fig. 17.** Effect of carbohydrate-restricted diets on glycosylated hemoglobin (HbA1c) in adults with diabetes in East-Asian countries (China, Japan, Taiwan). mLCD, moderately-low or low carbohydrate diet; WMD, weighted mean difference; CI, confidence interval.

**Supplementary Fig. 18.** Effect of carbohydrate-restricted diets on body weight in adults with diabetes. (A) Moderately-low or low carbohydrate diets (mLCD). (B) Very-low carbohydrate diets (VLCD). BW, body weight; WMD, weighted mean difference; CI, confidence interval.

(A)

(B)

**Supplementary Fig. 19.** Effect of carbohydrate-restricted diets on blood pressure in adults with diabetes. (A) Moderately-low or low carbohydrate diets (mLCD). (B) Very-low carbohydrate diets (VLCD). SBP, systolic blood pressure; WMD, weighted mean difference; CI, confidence interval; DBP, diastolic blood pressure.

(A)

**SBP (mmHg) / mLCD**

**DBP (mmHg) / mLCD**

(B)

**SBP (mmHg) / VLCD**

**DBP (mmHg) / VLCD**

**Supplementary Fig. 20.** Effect of carbohydrate-restricted diets on lipid profiles in adults with diabetes. (A) Moderately-low or low carbohydrate diets (mLCD). (B) Very-low carbohydrate diets (VLCD). TG, triglyceride; WMD, weighted mean difference; CI, confidence interval; HDL-C, high-density lipoprotein cholesterol; LDL-C, low-density lipoprotein cholesterol.

(A)

(B)

**Supplementary Fig. 21.** Effect of carbohydrate-restricted diets on fasting glucose in adults with diabetes. (A) Moderately-low or low carbohydrate diets (mLCD). (B) Very-low carbohydrate diets (VLCD). WMD, weighted mean difference; CI, confidence interval.

**Supplementary Fig. 22.** Effect of carbohydrate-restricted diets on insulin resistance (HOMA-IR) in adults with diabetes. (A) Moderately-low or low carbohydrate diets (mLCD). (B) Very-low carbohydrate diets (VLCD). WMD, weighted mean difference; CI, confidence interval.

**Supplementary Fig. 23.** Risk of bias assessment in studies evaluating the effects of carbohydrate-restricted diets in adults with hypertension.


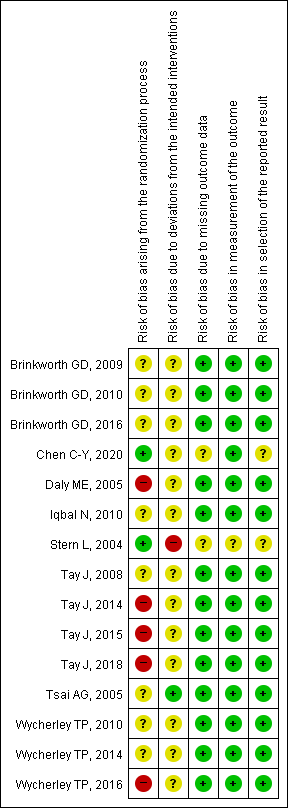

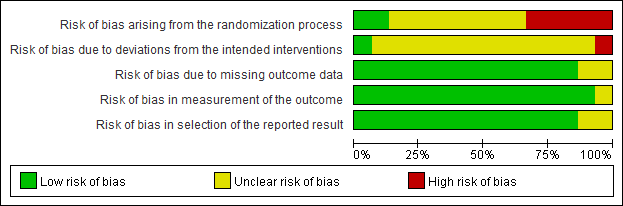


**Supplementary Fig. 24.** Effect of carbohydrate-restricted diets on body weight in adults with hypertension. (A) Moderately-low or low carbohydrate diets (mLCD). (B) Very-low carbohydrate diets (VLCD). BW, body weight; WMD, weighted mean difference; CI, confidence interval.

**Supplementary Fig. 25.** Effect of carbohydrate-restricted diets on lipid profile in adults with hypertension. (A) Moderately-low or low carbohydrate diets (mLCD). (B) Very-low carbohydrate diets (VLCD). TG, triglyceride; WMD, weighted mean difference; CI, confidence interval; HDL-C, high-density lipoprotein cholesterol; LDL-C, low-density lipoprotein cholesterol.
